# *ABCC1*, *ABCG2* and *FOXP3*: Predictive Biomarkers of Toxicity from Methotrexate Treatment in Patients Diagnosed with Moderate-to-Severe Psoriasis

**DOI:** 10.3390/biomedicines11092567

**Published:** 2023-09-19

**Authors:** Cristina Membrive-Jiménez, Sayleth Vieira-Maroun, Noelia Márquez-Pete, Yasmin Cura, Cristina Pérez-Ramírez, Jesús Tercedor-Sánchez, Alberto Jiménez-Morales, María del Carmen Ramírez-Tortosa

**Affiliations:** 1 Pharmacogenetics Unit, Pharmacy Service, University Hospital Virgen de las Nieves, 18014 Granada, Spainnoeliamarquezpete@gmail.com (N.M.-P.);; 2 Department of Biochemistry and Molecular Biology II, Faculty of Pharmacy, University of Granada, 18011 Granada, Spain; mramirez@ugr.es; 3 Dermatology Service, University Hospital Virgen de las Nieves, 18014 Granada, Spain; 4 Hospital Pharmacy Department, University Hospital Virgen de las Nieves, 18014 Granada, Spain

**Keywords:** psoriasis, pharmacogenetics, methotrexate, genetic polymorphisms, toxicity, adverse events, adverse drug reactions, hepatotoxicity

## Abstract

Background: Methotrexate (MTX) is one of the most extensively used drugs in the treatment of moderate-to-severe psoriasis (PS). However, it frequently must be suspended owing to the toxicity in certain patients. Objective: To evaluate the influence of *ABCC1, ABCG2*, and *FOXP3* in the development of MTX toxicity in PS. Methods: Retrospective cohort study with 101 patients. Five single-nucleotide polymorphisms (SNPs) were genotyped using real-time polymerase chain reaction with TaqMan probes. Results: Patients carrying *ABCC1* rs2238476-AG genotype (AG vs. GG: OR = 8.04; 95% CI = 1.48–46.78; *p* = 0.015); *FOXP3* rs376154-GT and GG genotypes (GT vs. TT/GG: OR = 3.86; 95% CI = 1.17–13.92; *p* = 0.031) and *ABCG2* rs13120400-T allele (T vs. CC: OR = 8.33; 95% CI = 1.24–164.79; *p* = 0.059) showed a higher risk of developing more than one adverse effect. The toxicity analysis by subtypes showed that the *ABCC1* rs2238476-AG genotype (AG vs. GG: OR = 8.10; 95% CI = 1.69–46.63; *p* = 0.011) and *FOXP3* rs376154-GT genotype (OR = 4.11; 95% CI = 1.22–15.30; *p* = 0.027) were associated with the appearance of asthenia. No association of the other ABCC1 polymorphisms (rs35592 and rs246240) with MTX toxicity was found. Conclusion: *ABCC1, ABCG2*, and *FOXP3* polymorphisms can be considered to be risk biomarkers of toxicities in PS patients treated with MTX.

## 1. Introduction

Psoriasis (PS) is a chronic, inflammatory, autoimmune disease affecting 1–3% of the population worldwide [1,2]. The main characteristic is the production of skin lesions, but it is also associated with potentially disabling pathologies, such as erectile dysfunction in 35% of patients and arthropathy in 40% [3,4,5,6,7,8]. Consequently, psoriasis is considered a systemic entity that severely impacts the quality of life of patients [9].

Etiology is not completely defined; however, it is believed that different factors are involved [10,11]. Regarding genetics, it has been observed that there are multiple chromosomal loci associated with susceptibility to psoriasis (PSORS), the most prominent being PSORS1, responsible for 50% of the heritability of the disease [12]. In addition, the incidence of psoriasis is different according to ethnicity, and higher among relatives and even more so among monozygotic twins [13]. The presence of the human leukocyte antigen HLA-Cw6 is particularly associated with phenotypic features, along with early development and course of the disease [14,15].

Genetic and environmental factors such as stress, infections, or unhealthy lifestyle habits predispose to the onset of the pathology, while abnormalities in cutaneous immune responses are responsible for the development and maintenance of psoriatic inflammation [10,14]. When plasmacytoid dendritic cells, keratinocytes, natural killer cells, and macrophages are activated they secrete various cytokines (tumor necrosis factor and Interleukin 1, respectively) that stimulate myeloid dendritic cells [16]. The activated dendritic cells promote the production of a cytokine cascade that activates keratinocyte proliferation in the epidermis. Consequently, hyperproliferation of keratinocytes in the epidermis and vascular endothelium occurs, resulting in epidermal hyperplasia typical of psoriatic lesions [11].

There are different types of psoriasis, but the most prevalent is “psoriasis vulgaris or plaque psoriasis” characterized by clinical manifestations in the form of erythematous plaques on the scalp, elbows, knees and back covered with whitish scales [17,18,19]. The severity of the lesions is measured with the indicators of psoriasis area severity index (PASI), body surface area (BSA), and dermatologic quality-of-life index (DLQI). When these indicators are greater than 10, moderate–severe psoriasis is considered [20]. The treatments employed are aimed at blocking the inflammatory response [21]. Pharmacological treatment is chosen according to the severity, in mild psoriasis the treatment is mainly based on topical and symptomatic therapy [22,23]. However, in cases of moderate–severe psoriasis, treatment with systemic therapy (methotrexate, cyclosporine, acitretin, apremilast, fumaric acid esters), phototherapy, or photochemotherapy is indicated [9,24,25]. In patients diagnosed with moderate–severe psoriasis who do not respond to systemic treatment or who are contraindicated, treatment with biologics is used [20]. Generally, the drug most frequently used in the treatment of moderate-to-severe PS is methotrexate (MTX) at low doses, because of the benefits it shows (proven effectiveness, low cost, relatively simple administration, and its usefulness in combination with other treatments) [26]. Furthermore, it can be administered orally or subcutaneously, the latter route being the more advantageous, with better absorption and greater bioavailability [27,28].

Methotrexate is an antimetabolite with antiproliferative, anti-inflammatory, and immunosuppressive activity. Although the mechanism of action at low doses is not clear, it is related to the ability to form intracellular polyglutamates and to increased adenosine formation due to inhibition of the ATIC (5-aminoimidazole-4-carboxamide ribonucleotide formyltransferase/IMP cyclohydrolase) enzyme, which leads to apoptosis in activated lymphocytes or to inhibition of the activation and expression of certain adhesion molecules in them [29,30,31]. Consequently, the action of MTX may be conditioned at various points on the metabolic pathway [32,33,34].

This drug has proved effective, reaching PASI75 and PASI90 after 16 and 24 weeks of treatment. However, it is frequently associated with reversible adverse effects (AEs) which entail temporary interruption of treatment or modification of the dose [35]. Long-term studies show that AEs occur in around 61–95% of patients [36]. The most common AEs are nausea, anorexia, and asthenia, depending on the dose and usually occurring at the start of therapy [25]. 

Moreover, prolonged use of MTX is associated with hepatotoxicity and hematological, renal, pulmonary, and cutaneous toxicity, among others [37]. Specifically, long-term treatment with MTX is associated with progressive and dose-dependent hepatotoxicity. Previous studies have observed that 23–33% of patients show an increase in liver enzyme levels; however, in the absence of excessive alcohol consumption, it rarely causes clinically significant liver damage [38]. They have also observed that it may cause hematological toxicity through hematopoietic suppression of MTX, specifically pancytopenia, which is reversible with dose reduction or temporary withdrawal of MTX [39,40]. In 3% of the patients studied, renal toxicity occurred in prolonged treatment with MTX [37,40]. Pulmonary toxicity has also been described as a risk associated with MTX treatment; in particular, there are reports of pulmonary fibrosis and pneumonitis [26]. Severe skin reactions were observed in patients with long-term MTX; in particular, 4% of the patients studied showed alopecia [40,41]. Finally, it is important to note the association of MTX treatment with a higher incidence of certain malignancies, such as lymphoma [41].

Despite the risks involved in using MTX, it has been observed that AEs limit treatment in only 6.9% of patients treated with MTX for 6 months, and therefore the risk-benefit profile of MTX is acceptable [42]. However, it is crucial to conduct exhaustive pharmacotherapeutic follow-ups of patients receiving treatment with MTX and to use folic acid supplementation [43].

In conclusion, although low-dose MTX treatment in patients diagnosed with moderate-to-severe PS is safe and effective, certain patients do not attain an optimum response or they experience various degrees of toxicity [44]. This variability in response and toxicity may be due to genetic factors. Interindividual variations in the genes implicated in the disease environment of the condition, pharmacokinetics, pharmacodynamics, metabolism, or in the mechanism of action of MTX may explain the emergence of adverse effects in certain patients treated with the same therapeutic conditions [45]. Specifically, genetic polymorphisms in the membrane efflux transporters responsible for the intracellular entry of MTX (*ABCC1-4, ABCB1*, and *ABCG2*), as well as other genes involved in the physiopathogeny of the disease have proved to play a crucial role in interindividual variability in response to and toxicity from MTX [46].

In particular, the *ABCC1* gene is located on chromosome 16 and encodes the ABCC1 protein, also known as multidrug-resistance-associated protein 1 (MRP1). It is found mainly in the basolateral plasma membranes of the enterocytes transporting endogenous substances, as well as xenobiotics and their metabolites [46,47,48]. Genetic polymorphisms in *ABCC1* (rs35592 T>C; rs246240 A>G, rs2238476 G>A) have been associated with response to and toxicity from MTX in psoriasis patients [49].

Similarly, the *ABCG2* gene, on chromosome 4, codes for a membrane transporter characterized by being essentially a xenobiotic transporter that plays an important part in resistance to numerous drugs, such as MTX. The ABCG2 protein transports MTX and its forms of polyglutamate, specifically polyglutamate 2 and 3, and therefore plays a key role in MTX’s mechanism of action [50]. Previous studies have found an association between response to and toxicity from MTX treatment in patients diagnosed with PS and the po-lymorphism of this gene (*ABCG2* rs13120400, C>T) [50]. Consequently, genetic alterations in *ABCC1* and *ABCG2* may modify the metabolism of MTX and lead to variability in the toxicity of this drug.

On the other hand, the *FOXP3* gene, located on the X chromosome, encodes a protein from the fork-winged helix family of transcription factors which plays an essential role in modulating regulatory T cells and can act as a transcriptional repressor or activator depending on its interactions with other transcription factors, histones, acetylases, and deacetylases [51,52]. Alterations in *FOXP3* (rs3761548; G>C/T/A) can potentiate the inflammatory cascade in psoriasis and consequently not respond to and/or provoke AEs due to MTX treatment [53,54].

Based on all the foregoing, the object of this study was to evaluate the influence of polymorphisms of the *ABCC1, ABCG2,* and *FOXP3* genes on the development of toxicity from treatment with MTX in moderate-to-severe psoriasis.

## 2. Materials and Methods

### 2.1. Study Design

Retrospective observational cohort study.

### 2.2. Study Subjects

The study included 101 Caucasian patients, over the age of 18, diagnosed with moderate-to-severe PS (BSA and PASI > 10), under treatment with MTX as monotherapy or in combination with biologic medications, for at least two months, from the Dermatology Department of the HUVN, during the period between January 2019 and November 2020. The starting dose of oral MTX was 15 mg/week, in combination with folic acid (5 mg/week, administered 24 h after the MTX), a dose approved in clinical practice guidelines [55]. 

### 2.3. Sociodemographic and Clinical Variables

The sociodemographic variables collected were sex, age at the start of MTX therapy, family history, and smoking and drinking status. We also collected data on the clinical features of the PS, including the type of psoriasis (plaque, pustular, inverse, guttate, or a combination of different types of psoriasis: plaque and guttate, plaque and pustular, plaque and inverse, plaque, guttate and inverse) and the location of the lesions (scalp and face, nails, palmoplantar, torso, and upper and lower extremities or flexures). In addition, we studied the development of psoriatic arthritis, concomitant diseases, therapy adherence, duration of MTX treatment, route of administration of MTX, concomitant medication, and maximum dose of MTX (mg/week).

The adverse reactions collected were classified as gastrointestinal toxicity (nausea, vomiting, diarrhea, and stomatitis), hepatotoxicity (appearance of abnormalities in hepatic parameters [ALT/AST, GGT, total bilirubin, procollagen peptide], exacerbation or reactivation of hepatitis, fibrosis, or cirrhosis), hematological toxicity (anemia, leukopenia, thrombocytopenia, and/or pancytopenia), asthenia, infections, neurological toxicity (dizziness, headache disorders), skin toxicity (hair loss, skin rash), nephrotoxicity (appearance of abnormalities in renal parameters [creatinine, urea, uric acid] or renal disorders).

### 2.4. Sample Processing and Genotyping

#### 2.4.1. DNA Isolation

DNA was obtained from saliva samples with buccal swabs (OCR-100 kit), after the inclusion of the patients and signing of the informed consent. Subsequently, DNA extraction was performed, using the QIAamp DNA Mini Kit (Qiagen GmbH, Hilden, Germany), following the manufacturer’s instructions for the purification of DNA from saliva, and stored at −20 °C. To check DNA concentration and purity values, a NanoDrop 2000 UV spectrophotometer was used with an absorbance ratio of 280/260 and 280/230.

#### 2.4.2. Detection of Gene Polymorphisms

The *ABCC1* rs35592 (TaqMan assay ID C___1003671_10), *ABCC1* rs246240 (assay ID C___1003698_10), *ABCC1* rs2238476 (assay ID C___16172578_10), *ABCG2* rs13120400 (assay ID C___9510480_10), and *FOXP3* rs3761548 (assay ID C___27476877_10) gene polymorphisms were determined by real-time polymerase chain reaction (PCR) using TaqMan^®^ probes (ABI Applied Biosystems, 7300 Real-Time PCR System, Foster City, CA, USA).

### 2.5. Toxicity Variables

Toxicity was evaluated according to the common terminology criteria for adverse events (CTCAE) using version 5.0 (U.S. Department of Health and Human Services (HHS), Washington DC, USA). The severity of the adverse events was classified as presence (toxicity grade 1–4) or absence (no presence of toxicity). General toxicity was defined as present when there was at least one adverse event (grade 1–4). The occurrence of more than one and more than two adverse events was also analyzed.

### 2.6. Statistical Analysis

Statistical analysis was performed using the freely available software R 3.5.1 (R Foundation for Statistical Computing, Vienna, Austria). Normal quantitative variables were expressed as mean (±standard deviation) and as median (p50) and percentiles (25 and 75) for non-normal variables. Normality analysis was performed by applying the Kolmogorov–Smirnov test. Pearson’s chi-square test was used for bivariate analysis between toxicity and polymorphisms. Fisher’s exact test was applied for associations between toxicity and qualitative variables, while Student’s *t*-test was used for normal quantitative variables, and the Mann–Whitney test for non-normal variables. The association with SNPs was evaluated in multiple models (genotypic, dominant, and recessive), which were defined as follows: genotypic (DD vs. Dd vs. dd), dominant ((DD, Dd) vs. dd) and recessive (DD vs. (Dd, dd)), where D is the major allele (wild-type) and d the minor allele (variant). 

The adjusted odds ratio (OR) and 95% confidence interval (95% CI) were obtained by logistic or linear regression from multivariate analysis. The Hosmer–Lemeshow test was applied to determine the goodness-of-fit of each model. In addition, the omnibus test and the Cox-Snell and Nagelkerke r2 coefficients were calculated. We considered a statistically significant probability with a value of 0.05 or less.

For the analysis of polymorphisms, haplotype frequencies were calculated, as well as Hardy-Weinberg equilibrium and linkage disequilibrium (Lewontin’s D (D’) and linkage disequilibrium coefficient (r2)). This genetic analysis was performed with the PLINK application for genome-wide association analysis and SNPstats, a web-based tool for association study analysis [56,57,58,59].

## 3. Results

### 3.1. Patient Characteristics

A total of 101 Caucasian patients diagnosed with moderate-to-severe PS under treatment with MTX were included in this study. As Table 1 shows, the median age of the patients at diagnosis was 27.25 (18.42–44.25) years, and the majority were women (52/101; 51.49%), non-smokers (49/101; 48.51%) and non-drinkers (61/101; 60.40%). The information on family history of PS was positive in 51.49% of the patients (52/101). A total of 73.27% of them had PS vulgaris with plaques (74/101), with the lesions located mainly on the torso and upper and lower extremities (93/101; 92.08%). Thirty-one patients developed psoriatic arthritis (31/101; 30.69%). The mean age of starting MTX therapy was 45.60 ± 14.79 years, and the median duration of MTX treatment was 15 [5,6,7,8,9,10,11,12,13,14,15,16,17,18,19,20,21,22,23,24,25,26,27,28,29,30,31,32,33] months. In most of the patients, MTX was orally administered (60/101; 59.41%), as monotherapy (93/101; 92.08%), with a median maximum dose of 12.5 [10.0–15.0] mg/week, and 69.31% of the patients adhered to the treatment (70/101).

The patients showed the following grade 1–4 toxicity values: 36.63% hepatotoxicity (37/101), 28.71% gastrointestinal toxicity (29/101), 27.72% asthenia (28/101), 8.91% neurotoxicity (9/101), 7.92% cutaneous toxicity (8/101), 5.94% infections (6/101), 2.97% hematological toxicity (3/101), and 0.99% nephrotoxicity (1/101). The clinical and sociodemographic characteristics are described in Table 1.

### 3.2. Influence of Clinical-Pathological Characteristics on Toxicity

#### 3.2.1. Global Toxicity

In the bivariate analysis a significant association was found between the presence of toxicity and the development of psoriatic arthritis (OR = 6.60, 95% CI = 2.08–29.44, *p* = 0.002; Table S1), and also with the MTX administration route (both routes: OR = 4.04, 95% CI = 1.28–15.53; subcutaneous: OR = 2.65, 95% CI = 1.02–7.78; *p* = 0.028; Table S1). In addition, a tendency was found toward an association between patients in whom PS was in the nails and the risk of toxicity from MTX (OR = 2.24, 95% CI = 0.89–5.81, *p* = 0.058; Table S1). In the multivariate analysis, no significant association was found between developing toxicity through MTX and the clinical and sociodemographic variables studied.

Furthermore, a significant association was found between the presence of more than one adverse effect and patients who were women (OR = 3.20, 95% CI = 1.37–7.82, *p* = 0.007; Table S2), ex-smokers (OR = 5.56, 95% CI = 1.67–20.48, *p* = 0.019; Table S2) and with psoriatic arthritis (OR = 3.26, 95% CI = 1.36–8.01, *p* = 0.007; Table S2). The MTX administration route was also associated with the risk of developing more than one adverse effect (both: OR = 13.88, 95% CI = 4.45–49.21; subcutaneous: OR = 3.81, 95% CI = 1.32–11.81; *p* < 0.001; Table S2). Moreover, a tendency was found toward an association between the presence of more than one adverse effect and that of inverse PS (OR = 2.31, 95% CI = 0.94–5.72, *p* = 0.062; Table S2), as well as with the longer duration of MTX therapy (OR = 1.02, 95% CI = 1.00–1.04, *p* = 0.063; Table S2). The multivariate analysis showed an association between the emergence of more than one adverse effect, development of psoriatic arthritis (OR = 3.49, 95% CI = 1.29–9.91, *p* = 0.015), and MTX administration route (both: OR = 14.67, 95% CI = 4.51–54.89, *p* < 0.001; subcutaneous: OR = 3.32; 95% CI = 1.09–10.68, *p* = 0.036) (details of these values are shown in Table 2).

The presence of more than two adverse effects during MTX therapy, after the bivariate analysis, was associated with female sex (OR = 4.16, 95% CI = 1.35–21.24, *p* = 0.017; Table S3) and the MTX administration route (both: OR = 6.04, 95% CI = 1.49–30.62; subcutaneous: OR = 2.93, 95% CI = 0.66–15.29; *p* = 0.038; Table S3). The multivariate analysis did not reveal an association between the presence of more than two adverse effects and the clinical-pathological variables studied.

#### 3.2.2. Toxicity Subtypes

In the bivariate analysis, no significant association was found between the emergence of hepatotoxicity and the clinical-pathological variables studied (Table S4). Conversely, the emergence of gastrointestinal toxicity was related to female sex (OR = 2.78, 95% CI = 1.14–7.20, *p* = 0.026; Table S5), smoking status (non-smoker: OR = 1.88, 95% CI = 0.62–6.45; ex-smoker: OR = 5.72, 95% CI = 1.66–22.34; *p* = 0.016; Table S5), alcohol (non-drinker: OR = 3.99, 95% CI = 1.46–12.96; ex-drinker: OR = 6.60, 95% CI = 0.24–186.55; *p* = 0.013; Table S5), and MTX administration route (subcutaneous: OR = 2.08, 95% CI = 0.66–6.68; both: OR = 8.00, 95% CI = 2.65–26.52; *p* = 0.001, Table S5). Moreover, a tendency was found toward an association between gastrointestinal toxicity and PS located in the nails (OR = 2.48, 95% CI = 1.00–6.64, *p* = 0.053; Table S5). Similarly, developing asthenia was related to the MTX administration route (*p* < 0.001), showing a higher risk in those who used both routes (OR = 9.93; 95% CI = 3.07–36.90) and those who used only the subcutaneous route (OR = 4.20; 95% CI = 1.31–15.04) (details of all these values are given in Table S6).

The bivariate analysis showed that the route of administration of MTX (subcutaneous: OR = 7.07, 95% CI = 0.98–142.41; both: OR = 9.19, 95% CI = 1.26–186.15; *p* = 0.047; Table S7), as well as the presence of inverse psoriasis (OR = 6.36, 95% CI = 1.55–32.17, *p* = 0.006; Table S7), was associated with a higher risk of showing neurotoxicity in our patients. In addition, the development of cutaneous adverse events was associated with patients suffering from psoriatic arthritis (OR = 8.16, 95% CI = 1.75–58.25, *p* = 0.009; Table S8). Similarly, the bivariate analysis revealed that patients with lesions on the scalp and face had a lower risk of developing infections (OR = 7.50, 95% CI = 1.36–56.97, *p* = 0.027; Table S9). No significant association was found between the emergence of hematological toxicity or nephrotoxicity and the clinical variables analyzed (Tables S10 and S11).

### 3.3. Influence of Genetic Polymorphisms on Toxicity

#### 3.3.1. Genotype Distribution

The distribution of all polymorphisms studied is consistent with that expected according to the Hardy-Weinberg equilibrium model (Supplementary Table S12). The linkage disequilibrium values D’ and r2 are presented in Supplementary Table S13. The minor allele frequencies of all polymorphisms were higher than 1% and therefore, none of them have been excluded from the analysis (Table S14). 

#### 3.3.2. Global Toxicity

The bivariate analysis showed that patients carrying the *ABCG2* rs13120400-TT and CT genotypes showed a higher risk of grade 1–4 toxicity during MTX therapy (TT vs. CC/CT: OR = 13.93, 95% CI = 2.02–279.52; CT vs. CC/TT: OR = 11.15, 95% CI = 1.59–225.34, *p* = 0.023; Table S15). Moreover, the *ABCG2* rs13120400-T allele emerged as the allele for risk of MTX toxicity in our patients (T vs. CC: OR = 12.59, 95% CI = 1.92–247.28, *p* = 0.012; Table S15). In addition, in the multivariate analysis a tendency was found towards association between patients carrying the *ABCG2* rs13120400-T allele (T vs. CC: OR = 8.33; 95% CI = 1.24–164.79; *p* = 0.059) with psoriatic arthritis (OR = 5.60; 95% CI = 1.74–25.18; *p* = 0.009) and the risk of developing toxicity during treatment with MTX (all these values are shown in Table 3).

In the study of the emergence of multiple adverse effects, the bivariate analysis showed an association between patients carrying the *FOXP3* rs3761548-GT and GG genotypes and a higher risk of having more than one adverse effect (GT vs. TT/GG: OR = 3.24, 95% CI = 1.20–9.15; GG vs. TT/GT: OR = 1.03, 95% CI = 0.36–2.91; *p* = 0.034; Table S16). The multivariate analysis showed an association between the *FOXP3* rs3761548-GT genotype (GT vs. TT/GG: OR = 3.86, 95% CI = 1.17–13.92, *p* = 0.031) and the risk of having more than one adverse effect, adjusted for MTX administration route (both: OR = 15.92, 95% CI = 4.60–64.78, *p* < 0.001; subcutaneous: OR = 4.07, 95% CI = 1.26–14.43, *p* = 0.022) and developing psoriatic arthritis (OR = 4.28, 95% CI = 1.48–13.45, *p* = 0.009) (all these values are shown in Table 4).

Similarly, a significant association was found between the presence of more than two adverse effects and the *ABCC1* rs2238476-AG genotype (OR = 4.85, 95% CI = 1.09–19.89, *p* = 0.039; Table S17) and *FOXP3* rs3761548-GT and GG genotypes (GT vs. TT/GG: OR = 5.55, 95% CI = 1.47–27.20; GG vs. TT/GT: OR = 1.28, 95% CI = 0.22–7.34, *p* = 0.021; Table S17). After the multivariate analysis was performed, it was confirmed that patients carrying the *ABCC1* rs2238476-AG genotype (AG vs. GG: OR = 8.04, 95% CI = 1.48–46.78, *p* = 0.015) and the *FOXP3* rs3761548-GT genotype (GT vs. TT/GG: OR = 7.48, 95% CI = 1.68–46.23, *p* = 0.014) showed a higher risk of suffering more than two adverse effects, adjusted for MTX administration route (both: OR = 7.32, 95% CI = 1.54–47.02, *p* = 0.018; subcutaneous: OR = 4.89, 95% CI = 0.95–32.46, *p* = 0.069) (all these values are shown in Table 5).

#### 3.3.3. Toxicity Subtypes

The bivariate analysis showed a tendency towards an association between the presence of the *ABCG2* rs13120400-TT genotype and the risk of developing hepatotoxicity during MTX treatment (C vs. TT: OR = 0.44, 95% CI = 0.19–1.02, *p* = 0.058; Table S18). As for the emergence of asthenia, patients carrying the *ABCC1* rs2238476-AG genotype showed a higher risk of having this adverse event during therapy with MTX (OR = 4.70, 95% CI = 1.23–19.87, *p* = 0.026; Table S20). Moreover, a tendency was found toward an association between the *FOXP3* rs3761548-GT and GG genotypes and asthenia during MTX therapy (GT vs. TT/GG: OR = 3.25, 95% CI = 1.14–9.79; GG vs. TT/GT: OR = 1.12, 95% CI = 0.35–3.53, *p* = 0.051; Table S20). The multivariate analysis, adjusted for MTX administration route, showed that the *ABCC1* rs2238476-AG genotype (AG vs. GG: OR = 8.10, 95% CI = 1.69–46.63, *p* = 0.011) and the *FOXP3* rs3761548-GT genotype (OR = 4.11, 95% CI = 1.22–15.30, *p* = 0.027) were associated with a higher risk of having asthenia during MTX therapy (these values are shown in Table 6). Conversely, the bivariate analysis of gastrointestinal toxicity risk and the polymorphisms studied revealed no significant results (Table S19).

In the bivariate analysis, a tendency was found toward an association between patients carrying the *ABCC1* rs2238476-AG genotype and the risk of nephrotoxicity (*p* = 0.099; Table S21). However, no association was found between the SNPs studied and the development of neurotoxicity, cutaneous and hematological toxicity, and infections (Tables S22–S25).

## 4. Discussion

Methotrexate is the most commonly used conventional systemic medication in patients diagnosed with moderate-to-severe psoriasis [55]. Despite its widespread use, great interindividual variability has been observed regarding the appearance of adverse effects, and this, to a great extent, limits the effectiveness of the therapy in these patients [60,61]. Single-nucleotide polymorphisms (SNPs) in genes for enzymes involved in the metabolism and mechanism of action of MTX have been proposed as a potential cause of this variability [62]. Particularly noteworthy among them are those involved in the transport of MTX across the cell membrane, which could determine the passage of MTX into and out of the cell, and thus its pharmacodynamics and pharmacokinetics [63]. In this study, 101 patients diagnosed with moderate-to-severe PS and treated with MTX, as monotherapy or combined with biologic therapy, were evaluated to determine the influence of SNPs in the *ABCC1*, *ABCG2*, and *FOXP3* genes on the presence of toxicity due to MTX.

Various sociodemographic, clinical, and genetic factors may influence pharmacological response to and toxicity from MTX [64,65]. In our study, the appearance of possible grade 1–4 adverse effects was associated with the development of psoriatic arthritis, after the diagnosis of psoriasis. In line with these results, a study conducted on 235 Asian patients (China) diagnosed with the psoriatic disease (PS = 107; psoriatic arthritis = 128), under treatment with MTX, found a higher incidence of adverse events such as dizziness (9.4% vs. 0.9%, *p* = 0.007), gastrointestinal symptoms (25.0% vs. 12.1%, *p* = 0.01), and hepatotoxicity (26.6% vs. 15.0%, *p* = 0.04) in patients with psoriatic arthritis than in those with PS [66]. Furthermore, the route of administration of MTX was also associated with a higher prevalence of toxicity in our patients. Specifically, patients to whom MTX was administered subcutaneously or those in whom the route of administration had been changed showed a higher risk of adverse effects appearing (asthenia, gastrointestinal toxicity, and neurotoxicity) during MTX therapy, According to our results, a study conducted in 291 Caucasian patients (Netherlands) diagnosed with rheumatoid or psoriatic arthritis and under therapy with MTX at low doses showed a higher prevalence of intolerance in patients with parenteral administration of MTX (parenteral [20.6%] vs. oral [6.2%], *p* < 0.001) [67]. In contrast to these results, a study conducted in 28 Caucasian patients (Egypt) diagnosed with psoriasis and under treatment with oral (*n* = 14) and subcutaneous (*n* = 14) MTX showed a higher prevalence of gastrointestinal toxicity (71.4% vs. 14.3%) and hepatotoxicity (21.3% vs. 7.1%) in patients with oral administration of MTX [68]. An investigation conducted in 55 Caucasian patients (Czech Republic) diagnosed with juvenile idiopathic arthritis and treated with MTX found no significant differences between the route of administration of MTX and the risk of adverse effects (parenteral vs. oral, *p* = 0.236) [69].

In our study, female sex was associated with a higher risk of undergoing adverse events, specifically gastrointestinal toxicity. A study carried out on 809 Caucasian patients diagnosed with psoriasis (*n* = 690) and rheumatoid arthritis (*n* = 119) and treated with MTX also found a higher risk of hepatotoxicity in women (hazard ratio [HR] = 1.46, *p* < 0.001) [70]. Similarly, smoking and drinking status were found to be associated with a higher risk of gastrointestinal toxicity. In line with this, previous studies have highlighted the influence of alcohol and tobacco consumption on the mechanism of action of MTX, since they could interfere with the mechanism of transporting the drug towards the cell interior, therefore modifying the pharmacological response and producing toxicity [71].

Previous studies have revealed the influence of various SNPs on the toxicity of MTX in patients with autoimmune diseases, such as *MTHFR, MTR, ABCC1, ABCC2,* and *ABCG2* [72,73,74]. However, the existing evidence on the influence of genetics on MTX toxicity in patients with PS is limited. In our study, we found an association between the *ABCG2* rs13120400 SNP and the appearance of adverse effects after administration of MTX. Specifically, following the multivariate analysis, the *ABCG2* rs13120400-T allele was found to be associated with the risk of grade 1–4 toxicity, adjusted for the development of psoriatic arthritis. Similarly, patients carrying the *ABCG2* rs13120400-TT genotype showed a higher risk of hepatotoxicity from MTX. In line with our results, Warren et al. carried out a study in 374 Caucasian patients (United Kingdom) diagnosed with moderate-to-severe PS and treated with MTX, finding an association between the *ABCG2* rs13120400-TT genotype and lesser effectiveness of MTX (TT vs. CC/CT, *p* = 0.03) [49]. However, its association with toxicity from the drug was not evaluated [49]. The transporter ABCG2 is composed of six transmembrane domains and is in hepatocytes, enterocytes, and renal cells, among others [63,75]. Previous research has suggested that ABCG2 is involved in the transport of MTX and its toxic metabolite, 7-hydroxymethotrexate (7OH-MTX), in the liver and kidney, finding that SNPs in these genes could cause variations in the activity of ABCG2, contributing to toxicity in patients treated with MTX [76,77,78]. Specifically, the *ABCG2* rs13120400 SNP is encoded in intronic region 9 of the gene and its biological effect has not been studied to date [79]. This SNP could influence the transcription and translation processes of the ABCG2 protein, giving rise to a non-functional molecule and affecting the cellular transport of MTX [79].

Another of the SNPs evaluated in this study was *ABCC1* rs2238476, located in intron 23 of the *ABCC1* gene, which codes for the membrane transporter protein ABCC1, responsible for the transport of MTX, among other drugs [49]. The main task of ABCC1 is to protect cells against toxic effects and contribute to the barrier function [80]. In our study, patients carrying the *ABCC1* rs2238476-AG genotype showed a higher risk of grade 1–4 adverse effects of MTX. In addition, this genotype was associated with the development of asthenia, after the multivariate analysis, as well as the appearance of nephrotoxicity in our patients. In line with this, a study with 374 Caucasian patients (United Kingdom) diagnosed with PS and under treatment with MTX related to the *ABCC1* rs2238476-GG genotype with a higher risk of emergence of toxicity in general (OR = 2.49; IC95% = 1.1–6.0; *p* = 0.01) [49]. This SNP could affect the protective function of the ABCC1 protein, modifying the passage of MTX out of the cells and therefore causing an intracellular accumulation of the drug, giving rise to various adverse reactions [79]. The association of these SNPs with mRNA/protein expression has been shown in some studies. A lower expression of ABCC1 could be the cause of greater toxicity due to the accumulation of MTX inside the cells [79].

We also found a statistically significant association between the *FOXP3* rs376154 SNP and the risk of toxicity from MTX. In the multivariate analysis, it was observed that patients carrying the *FOXP3* rs376154-GT and GG genotypes showed a higher risk of developing adverse events after the administration of MTX, specifically asthenia. Although there are no previous studies that evaluate the association of this SNP with the development of toxicity due to MTX, its influence on the efficacy of MTX has been assessed. A study conducted in 132 Caucasian patients (India) diagnosed with moderate-to-severe PS receiving MRX therapy and 57 healthy controls associated the *FOXP3* rs376154-GG and GT polymorphism with a lesser response to the drug (OR = 3.21, 95% CI = 1.50–6.87, *p* = 0.003) [81]. On the other hand, Wu Zhuo et al. carried out a study in 114 Asian patients (China) who had undergone a kidney transplant and were being treated with tacrolimus, finding that those carrying the FOXP3 rs376154-GT and TT genotypes showed a higher risk of nephrotoxicity from the drug (GT/TT vs. GG: HR = 10.71, 95% CI = 2.219–51.72, *p* = 0.036) [82]. The *FOXP3* rs376154 SNP is in the promoter region of the gene, and therefore a base change may modify the place where the transcription factors bind to the promoter, leading to defective transcription of the FOXP3 protein [83]. In addition, the *FOXP3* gene participates in the development and regulation of regulatory T cells and may contribute to the emergence of toxicity due to MTX via the inflammatory response suppression pathway mediated by IL-10 [82,84,85,86].

Regarding the *ABCC1* rs35592 and *ABCC1* rs246240 SNPs, no significant association was observed with the variables included in our study. These results are in line with those obtained in a study with 200 Caucasian patients (Australia) diagnosed with rheumatoid arthritis and treated with MTX at low doses, where no association was found between the *ABCC1* rs35592 SNP and the presence of toxicity (*p* > 0.05) [87]. Similarly, D’Cruz et al. studied the association between the *ABCC1* rs246240 SNP and plasma levels of MTX and its toxic metabolite 7OH-MTX in 100 Caucasian patients (United Kingdom) diagnosed with rheumatoid arthritis under treatment with MTX, without finding any significant association [88]. Conversely, Warren et al. conducted a study in 374 Caucasian patients (United Kingdom) diagnosed with moderate-to-severe PS receiving MTX therapy at low doses, finding that the patients who carried the *ABCC1* rs246240-AA genotype showed a higher risk of hepatotoxicity and gastrointestinal toxicity (OR = 2.2, 95% CI = 1.3–3.6, *p* = 0.001) [49].

Among the main limitations of our study, we should highlight the limited sample size, which may be responsible for the fact that no statistical association was found between the *ABCC1* rs35592 and *ABCC1* rs246240 SNPs and toxicity. Despite this, we obtained important and representative results that show the influence that certain SNPs have on the emergence of toxicity during the use of MTX in patients with moderate-to-severe PS. Thus, our sample size has a statistical power to detect genetic association of 57.44%. It is also worth emphasizing that this study was conducted with patients from the same hospital, all diagnosed with moderate-to-severe PS, according to the same therapeutic protocols, by the same team of dermatologists. This allows a high degree of homogeneity in the cohort, as well as in the collection of variables. 

Further studies with larger cohorts will be required to corroborate the prognostic value of biomarkers, in particular the ABCC1, ABCG2, and FOXP3 gene polymorphisms.

These results suggest that polymorphisms in the genes encoding the enzymes that participate in the cellular transport of MTX, particularly the *ABCG2* rs13120400, *ABCC1* rs2238476, and *FOXP3* rs376154 polymorphisms, could act as risk biomarkers for the emergence of adverse effects in patients with moderate-to-severe PS treated with MTX at low doses.

## 5. Conclusions

Our results suggest that patients with moderate-to-severe PS receiving MTX therapy showed a higher risk of having more than one adverse effect when they carried the *ABCC1* rs2238476-AG and *FOXP3* rs376154-GT and GG genotypes and the *ABCG2* rs13120400-T allele. The analysis of toxicity by subtypes also revealed that the *ABCC1* rs2238476-AG and *FOXP3* rs376154-GT genotypes were associated with the presence of asthenia, the *ABCG2* rs13120400-TT genotype was associated with grade 1–4 hepatotoxicity, and patients carrying the *ABCC1* rs2238476-AG genotype showed a tendency towards association with the emergence of grade 1–4 nephrotoxicity. No influence of the *ABCC1* rs35592 and *ABCC1* rs246240 SNPs on the appearance of MTX toxicity was found in our patients. As regards the clinical variables, the risk of toxicity from MTX was associated with the development of psoriatic arthritis, the MTX administration route, the patient’s sex, and tobacco and alcohol consumption.

## Figures and Tables

**Table 1:** Clinicopathological characteristics of the 101 patients with moderate–severe psoriasis treated with methotrexate.

Variable	*n*	%	Media ± SD
Gender			
Female	52	51.49	-
Male	49	48.51	-
Age diagnosis PS	101	-	27.25 (18.42–44.25)
Family history PS	52	51.49	
Smoking			
Smoker	31	30.69	-
Non-smoker	49	48.51	-
Former Smoker	21	20.79	-
Alcoholic drinking			
Drinker	38	37.62	-
Non-drinker	61	60.40	-
Former drinker	2	1.98	-
Type of PS			
Plaque	74	73.27	-
Pustular	5	4.95	-
Inverse	1	0.99	-
Guttate	5	4.95	-
Plaque and Guttate	12	11.88	-
Plaque and Inverse	2	1.98	-
Plaque and pustular	1	0.99	-
Plaque, guttate and inverse	1	0.99	-
Location of lesions			
Trunk and lower and upper limbs	93	92.08	-
Scalp and face	77	76.24	-
Nails	58	57.43	-
Palmoplantar	19	18.81	-
Flexures	28	27.72	-
Psoriatic Arthritis	31	30.69	-
Comorbidities	57	56.44	-
Age of onset of MTX	101	-	45.60 ± 14.79
Duration of MTX treatment (months)	101	-	15 (5–33)
Administration type of MTX			
Oral	47	46.53	-
Subcutaneous	30	29.70	-
Both	24	23.76	-
Type of MTX therapy			
Monotherapy	93	92.08	-
Combination Therapy	8	7.92	-
Maximum MTX dose (mg/week)	101	-	12.5 (10–15)
Medication Adherence			
Adherent	70	69.31	-
Non-adherent	31	30.69	-
Toxicity (Grade 1–4)	69	68.32	-
Gastrointestinal toxicity (Grade 1–4)	29	28.71	-
Hepatotoxicity (Grade 1–4)	37	36.63	-
Hematological toxicity (Grade 1–4)	3	2.97	-
Nephrotoxicity (Grade 1–4)	1	0.99	-
Asthenia (Grade 1–4)	28	27.72	-
Nervous system toxicity (Grade 1–4)	9	8.91	-
Skin Toxicity (Grade 1–4)	8	7.92	-
Infections (Grade 1–4)	6	5.94	-
Occurrence of adverse events			-
More than 1 (grade 1–4)	36	35.64	-
More than 2 (grade 1–4)	15	14.85	-

Qualitative variables: frequency (percentage, %). Quantitative variables: Normal distribution: mean ± standard deviation (SD). Non-normal distribution: P50 (P25–P75).

**Table 2:** Multivariate regression analysis for the presence of more than adverse events based on clinical characteristics and genetic variables.

	Occurrence of Adverse Events	
	OR (CI_95%_)	*p*-Value
	>1 Adverse Event	
Development of psoriatic arthritis (yes)	3.49 (1.29–9.91)	0.015
MTX administration		
Subcutaneous	3.32 (1.09–10.68)	0.036
Both	14.67 (4.50–54.89)	<0.001

**Table 3:** Multivariate regression analysis for overall toxicity based on clinical characteristics and genetic variables.

	Overall Toxicity	
	OR (CI_95%_)	*p*-Value
Development of psoriatic arthritis (yes)	5.60 (1.74–25.18)	0.009
*ABCG2* rs13120400-T (T vs. CC)	8.33 (1.24–164.79)	0.059

**Table 4:** Multivariate regression analysis for the presence of more than one adverse event based on clinical characteristics and genetic variables.

	More than 1 Adverse Event	
	OR (CI_95%_)	*p*-Value
Development of psoriatic arthritis (yes)	4.28 (1.48–13.45)	0.009
**MTX administration**		
Both	15.92 (4.60–64.78)	<0.001
Subcutaneous	4.07 (1.26–14.43)	0.022
*FOXP3* rs3761548 (GT vs. TT/GG)	3.86 (1.17–13.92)	0.031

**Table 5:** Multivariate regression analysis for the presence of more than 2 adverse events based on clinical characteristics and genetic variables.

	More than 2 Adverse Events	
	OR (CI_95%_)	*p*-Value
MTX administration		
Both	7.35 (1.54–47.02)	0.018
Subcutaneous	4.89 (0.95–32.46)	0.069
*ABCC1* rs2238476 (AG vs. GG)	8.04 (1.48–46.78)	0.015
*FOXP3* rs3761548 (GT vs. TT/GG)	7.48 (1.68–46.23)	0.014

**Table 6:** Multivariate regression analysis for asthenia based on clinical characteristics and genetic variables.

	Asthenia	
	OR (CI_95%_)	*p*-Value
MTX administration		
Subcutaneous	6.45 (1.78–28.51)	0.007
Both	13.01 (3.49–60.27)	<0.001
*ABCC1* rs2238476-AG (AG vs. GG)	8.10 (1.69–46.63)	0.011
*FOXP3* rs3761548-GT (GT vs. TT/GG)	4.10 (1.22–15.30)	0.027

## Data Availability

Data unavailable due to privacy and ethical restrictions.

## Supplementary Materials

The following supporting information can be downloaded at: https://www.mdpi.com/article/10.3390/biomedicines11092567/s1, Table S1: Clinical variables and overall toxicity; Table S2: Clinical variables and more than 1 adverse event; Table S3: Clinical variables and more than 2 adverse events; Table S4: Clinical variables and hepatotoxicity; Table S5: Clinical variables and gastrointestinal toxicity; Table S6: Clinical variables and asthenia; Table S7: Clinical variables and neurotoxicity; Table S8: Clinical variables and skin toxicity; Table S9: Clinical variables and infections; Table S10: Clinical variables and hematologic toxicity; Table S11: Clinical variables and nephrotoxicity; Table S12: Hardy-Weinberg equilibrium; Table S13: Linkage disequilibrium; Table S14: Minor allele frequencies of SNPs; Table S15: SNP and overall toxicity; Table S16: SNP and more than 1 adverse event; Table S17: SNP and more than 2 adverse events; Table S18: SNP and hepatotoxicity; Table S19: SNP and gastrointestinal toxicity; Table S20: SNP and asthenia; Table S21: SNP and nephrotoxicity; Table S22: SNP and neurotoxicity; Table S23: SNP and cutaneous toxicity; Table S24: SNP and hematological toxicity; Table S25: SNP and infections.
