# Supplementary material for: ABCC1, ABCG2 and FOXP3: Predictive Biomarkers of Toxicity from Methotrexate Treatment in Patients Diagnosed with Moderate-to-Severe Psoriasis
Source: Biomedicines. 2023 Sep 19;11(9):2567. doi: 10.3390/biomedicines11092567 (PMC10526923; doi:10.3390/biomedicines11092567)
Supplement: Supplementary file 1 [file biomedicines-11-02567-s001.zip › Table S1.Clinical variables and overall toxicity.pdf]

Table S1. Clinical variables and overall toxicity

Characteristics	N	Overall toxicity		$χ^2$	p-value	OR	$IC_{95\%}$
		No<br>N (%)	Yes<br>(Grade 1-4)<br>N (%)				
**Gender**							
Female	52	15 (28.8)	37 (71.2)	0.399	0.528	-	-
Male	49	17 (34.7)	32 (65.3)				
**Age diagnosis PS**	101	26.5<br>(18.2-49.3)	28.5<br>(18.8-41.4)	0.702	0.486	-	-
**Family History of Ps**							
Yes	52	17 (32.7)	35 (67.3)	0.050	0.822	-	-
No	49	15 (30.6)	34 (69.4)				
**Smoking**							
Smoker	31	13 (41.9)	18 (58.1)	2.326	0.313	-	-
Non-smoking	49	14 (28.6)	35 (71.4)				
Former Smoker	21	5 (23.8)	16 (76.2)				
**Alcoholic drinking**							
Drinker	38	16 (42.1)	22 (57.9)	-	0.134	-	-
Non-drinker	61	15 (24.6)	46 (75.4)				
Former Drinker	2	1 (50.0)	1 (50.0)				
**Type of Psoriasis**							
Plaque	74	27 (36.5)	47 (63.5)	-	0.633*	-	-
Pustular	5	1 (20.0)	4 (80.0)				
Inverse	1	0 (0.0)	1 (100.0)				
Guttate	5	1 (20.0)	4 (80.0)				
Plaque and guttate	12	2 (16.7)	10 (83.3)				
Plaque and inverse	2	0 (0.0)	2 (100.0)				
Plaque and pustular	1	1 (100.0)	0 (0.0)				
Plaque, guttate and inverse	1	0 (0.0)	1 (100.0)				
**Localization**							
Trunk and limbs							
Yes	93	29 (31.2)	64 (68.8)	-	0.706*	-	-
No	8	3 (37.5)	5 (62.5)				
Scalp and face							
Yes	77	25 (32.5)	52 (67.5)	0.092	0.762	-	-
No	24	7 (29.2)	17 (70.8)				
Nails							
Yes	58	14 (24.1)	44 (75.9)	3.583	**0.058**	**2.24**	**0.89-5.81**
No	43	18 (41.9)	25 (58.1)			**1**	**-**
Palmoplantar							
Yes	19	7 (36.8)	12 (63.2)	0.288	0.592	-	-
No	82	25 (30.5)	57 (69.5)				
Flexures							
Yes	28	8 (28.6)	20 (71.4)	0.173	0.677	-	-
No	73	24 (32.9)	49 (67.1)				
**Development of PSA**							
Yes	31	3 (9.7)	28 (90.3)	10.007	**0.002**	**6.60**	**2.08-29.44**
No	70	29 (41.4)	41 (58.6)			**1**	**-**
**Comorbidities**							
Yes	57	14 (24.6)	43 (75.4)	3.066	0.079	-	-
No	44	18 (40.9)	26 (59.1)				
**Age of onset of MTX**	101	46.1916.13±	45.3314.25±	-	0.798	-	-
**MTX therapy duration (months)**	101	14.5<br>(8.75-23.25)	15.0<br>(5.0-37.0)	-	0.345	-	-

**MTX Administration**							
Oral	47	21 (44.7)	26 (55.3)	**7.135**	**0.028**	**1**	**-**
Subcutaneous	30	7 (23.3)	23 (76.7)			**2.65**	**1.02-7.78**
Both	24	4 (16.7)	20 (83.3)			**4.04**	**1.28-15.53**
**Type of MTX therapy**							
Monotherapy	93	30 (32.3)	63 (67.7)	-	1*	-	-
Combination Therapy	8	2 (25.0)	6 (75.0)				
**Maximum MTX dose (mg/week)**	101	12.5 (10.0-15.0)	15.0 (10.0-15.0)	-	0.551	-	-
**Therapeutic adherence**							
Adherent	70	24 (34.3)	46 (65.7)	-	0.664*		
Intentional non-adherent	30	8 (26.7)	22 (73.3)				
Unintentional non-adherent	1	0 (0.0)	1 (100.0)				

*p-value for the Fisher's test. PS: psoriasis; PSA: psoriatic arthritis
