# Supplementary material for: ABCC1, ABCG2 and FOXP3: Predictive Biomarkers of Toxicity from Methotrexate Treatment in Patients Diagnosed with Moderate-to-Severe Psoriasis
Source: Biomedicines. 2023 Sep 19;11(9):2567. doi: 10.3390/biomedicines11092567 (PMC10526923; doi:10.3390/biomedicines11092567)
Supplement: Supplementary file 1 [file biomedicines-11-02567-s001.zip › Table S10. Clinical variables and hematologic toxicity.pdf]

Table S10. Clinical variables and hematologic toxicity

Characteristics	N	Hematologic toxicity		X^2	p-valor	OR	$IC_{95\%}$
		NO N (%)	YES (Grade 1-4) N (%)				
**Gender**	101						
Female	52	50(96.2)	2(3.8)	-	1*	-	-
Male	49	48(98.0)	1(2.0)				
**Age diagnosis PS**	101	27.1 (18.0-44.3)	29.6 (24.2-34.3)	-	0.777	-	-
**Family History of Ps**	101						
Yes	52	49 (94.2)	3 (5.8)	-	0.243*	-	-
No	49	49 (100.0)	0 (0.0)				
**Smoking**	101						
Smoker	31	31 (100.0)	0 (0.0)	-	0.305*	-	-
Non-smoking	49	46 (93.9)	3 (6.1)				
Former Smoker	21	21 (100.0)	0 (0.0)				
**Alcoholic drinking**	101						
Drinker	38	38 (100.0)	0 (0.0)	-	0.325*	-	-
Non-drinker	61	58 (95.1)	3 (4.9)				
Former Drinker	2	2 (100.0)	0 (0.0)				
**Type of Psoriasis**	101						
Plaque	74	72(97.3)	2(2.7)	-	0.417*	-	-
Pustular	5	5(100.0)	0(0.0)				
Inverse	1	1(100.0)	0(0.0)				
Guttate	5	4(80.0)	1(20.0)				
Plaque and guttate	12	12(100.0)	0(0.0)				
Plaque and inverse	2	2(100.0)	0(0.0)				
Plaque and pustular	1	1(100.0)	0(0.0)				
Plaque, guttate and inverse	1	1(100.0)	1(0.0)				
**Localization**							
Trunk and limbs	101						
Yes	93	90(96.8)	3(3.2)	-	1*	-	-
No	8	8(100.0)	0(0.0)				
Scalp and face	101						
Yes	77	74(96.1)	3(3.9)	-	1*	-	-
No	24	24(100.0)	0(0.0)				
Nails	101						
Yes	58	56(96.6)	2(3.4)	-	1*	-	-
No	43	42(97.7)	1(2.3)				
Palmoplantar	101						
Yes	19	19(100.0)	0(0.0)	-	1*	-	-
No	82	79(96.3)	3(3.7)				
Flexures	101						
Yes	28	27 (96.4)	1 (3.6)	-	1*	-	-
No	73	71 (97.3)	2 (2.7)				
**Development of PSA**	101						
Yes	31	29(93.5)	2(6.5)	-	1*	-	-
No	70	69(98.6)	1(1.4)				
**Comorbidities**	101						

Yes	57	55(96.4)	2(3.5)	-	1*	-	-
No	44	43(97.7)	1(2.3)				
**Age of onset of MTX**	101	45.77±14.94	40.33±8.50	-	0.385	-	-
**MTX therapy duration (months)**	101	14.5 (5.0-35.3)	15.0 (8.5-19.5)	-	0.253	-	-
**MTX Administration**	101						
Oral	47	46 (97.9)	1 (2.1)	-	1*	-	-
Subcutaneous	30	29 (96.7)	1 (3.3)				
Both	24	23 (95.8)	1 (4.2)				
**Type of MTX therapy**	101						
Monotherapy	93	90 (96.8)	3 (3.2)	-	1*	-	-
Combination Therapy	8	8 (100.0)	0 (0.0)				
**Maximum MTX dose (mg/week)**	101	12.5 (10.-15.0)	15.0 (10.0-15.0)	-	0.756	-	-
**Therapeutic adherence**							
Adherent	70	68 (97.1)	2 (2.9)	-	1*	-	-
Intentional non-adherent	30	29 (96.7)	1 (3.3)				
Unintentional non-adherent	1	1 (100.0)	0 (0.0)				
*p-value for the Fisher's test. PS: psoriasis; PSA: psoriatic arthritis							
