# Supplementary material for: ABCC1, ABCG2 and FOXP3: Predictive Biomarkers of Toxicity from Methotrexate Treatment in Patients Diagnosed with Moderate-to-Severe Psoriasis
Source: Biomedicines. 2023 Sep 19;11(9):2567. doi: 10.3390/biomedicines11092567 (PMC10526923; doi:10.3390/biomedicines11092567)
Supplement: Supplementary file 1 [file biomedicines-11-02567-s001.zip › Table S11. Clinical variables and nephrotoxicity.pdf]

Table S11. Clinical variables and nephrotoxicity

Characteristics	N	Nephrotoxicity NO N (%)	Nephrotoxicity YES (Grade 1-4) N (%)	X^2	p-valor	OR	$IC_{95\%}$
**Gender**	101						
Female	52	51(98.1)	1(1.9)	-	**1***	**-**	**-**
Male	49	49(100.0)	0(0.0)				
**Age diagnosis PS**	101	27.1 (18.3-43.6)	48.8 (48.8-48.8)	-	1*	-	-
**Family History of Ps**	101						
Yes	52	52 (100.0)	0 (0.0)	-	0.485*	-	-
No	49	48 (98.0)	1 (2.0)				
**Smoking**	101						
Smoker	31	31 (100.0)	0 (0.0)	-	1*	**-**	**-**
Non-smoking	49	48 (92.0)	1 (2.0)				
Former Smoker	21	21 (100.0)	0 (0.0)				
**Alcoholic drinking**	101						
Drinker	38	38 (100.0)	0 (0.0)	-	1*	-	-
Non-drinker	61	60 (98.4)	1 (1.6)				
Former Drinker	2	2 (100.0)	0 (0.0)				
**Type of Psoriasis**	101						
Plaque	74	73(98.6)	1(1.4)	-	1*	-	-
Pustular	5	5(100.0)	0(0.0)				
Inverse	1	1(100.0)	0(0.0)				
Guttate	5	5(100.0)	0(0.0)				
Plaque and guttate	12	12(100.0)	0(0.0)				
Plaque and inverse	2	2(100.0)	0(0.0)				
Plaque and pustular	1	1(100.0)	0(0.0)				
Plaque, guttate and inverse	1	1(100.0)	0(0.0)				
**Localization**							
Trunk and limbs	101						
Yes	93	92 (98.9)	1(1.1)	-	1*	-	-
No	8	8(100.0)	0(0.0)				
Scalp and face	101						
Yes	77	76(98.7)	1(1.3)	-	1*	-	-
No	24	24(100.0)	0(0.0)				
Nails	101						
Yes	58	57(98.3)	1(1.7)	-	1*	**-**	
No	43	43(100.0)	0(0.0)				
Palmoplantar	101						
Yes	19	19(100.0)	0(0.0)	-	1*	-	-
No	82	81(98.8)	1(1.2)				
Flexures	101						
Yes	28	28 (100.0)	0 (0.0)	-	1*	**-**	**-**
No	73	72 (98.6)	1 (1.4)				
**Development of PSA**	101						
Yes	31	30(96.8)	1(3.2)	-	0.307*	**-**	**-**
No	70	70(100.0)	0(0.0)				
**Comorbidities**	101						
Yes	57	56(98.2)	1(1.8)	-	1*	-	-
No	44	44(100.0)	0(0.0)				
**Age of onset of MTX**	101	45.47±14.81	59.00±0.00	-	1*	-	-

**MTX therapy duration (months)**	101	14.5 (5.0-31.5)	144.0 (144.0-144.0)	-	1*	-	-
**MTX Administration**	101						
Oral	47	47 (100.0)	0 (0.0)	-	0.238*	-	-
Subcutaneous	30	30 (100.0)	0 (0.0)				
Both	24	23 (95.8)	1 (4.2)				
**Type of MTX therapy**	101						
Monotherapy	93	93 (100.0)	0 (0.0)	-	**0.079***	-	-
Combination Therapy	8	7 (87.5)	1 (12.5)				
**Maximum MTX dose (mg/week)**	101	12.5 (10.0-15.0)	20.0 (20.0-20.0)	-	1*	-	-
**Therapeutic adherence**							
Adherent	70	70 (100.0)	0 (0.0)	-	0.307*	-	-
Intentional non-adherent	30	29 (96.7)	1 (3.3)				
Unintentional non-adherent	1	1 (100.0)	0 (0.0)				

*p-value for the Fisher's test. PS: psoriasis; PSA: psoriatic arthritis.
