# Supplementary material for: ABCC1, ABCG2 and FOXP3: Predictive Biomarkers of Toxicity from Methotrexate Treatment in Patients Diagnosed with Moderate-to-Severe Psoriasis
Source: Biomedicines. 2023 Sep 19;11(9):2567. doi: 10.3390/biomedicines11092567 (PMC10526923; doi:10.3390/biomedicines11092567)
Supplement: Supplementary file 1 [file biomedicines-11-02567-s001.zip › Table S12. Hardy-Weinberg equilibrium.pdf]

Table S12. *Hardy-Weinberg Equilibrium*

Chr	SNP	Minor Allele	Major Allele	Genotype counts	Observed heterozygosity	Expected heterozygosity	p-value
4	rs13120400	C	T	6/42/53	0.4158	0.3917	0.6205
16	rs35592	C	T	3/40/58	0.396	0.3517	0.2678
16	rs2238476	A	G	0/10/91	0.09901	0.09411	1
16	rs246240	G	A	3/24/74	0.2376	0.2529	0.4501
23	rs3761548	G	T	12/28/12	0.5385	0.5	0.7813

Chr: Chromosome; SNP: single nucleotide polymorphism
