# Supplementary material for: ABCC1, ABCG2 and FOXP3: Predictive Biomarkers of Toxicity from Methotrexate Treatment in Patients Diagnosed with Moderate-to-Severe Psoriasis
Source: Biomedicines. 2023 Sep 19;11(9):2567. doi: 10.3390/biomedicines11092567 (PMC10526923; doi:10.3390/biomedicines11092567)
Supplement: Supplementary file 1 [file biomedicines-11-02567-s001.zip › Table S13.Linkage desequilibrium.pdf]

Table S13. Linkage disequilibrium

Chr	BP	SNP	Chr	BP	SNP	R^2	D’
16	16047966	rs35592	16	16025167	rs246240	0.2534	0.6545
Chr: Chromosome; BP: Physical position (base-pair)							
