# Supplementary material for: ABCC1, ABCG2 and FOXP3: Predictive Biomarkers of Toxicity from Methotrexate Treatment in Patients Diagnosed with Moderate-to-Severe Psoriasis
Source: Biomedicines. 2023 Sep 19;11(9):2567. doi: 10.3390/biomedicines11092567 (PMC10526923; doi:10.3390/biomedicines11092567)
Supplement: Supplementary file 1 [file biomedicines-11-02567-s001.zip › Table S14. Minor allele frequencies of SNPs.pdf]

Table S14. Minor allele frequencies of SNPs.

Chr	SNP	Minor Allele	Major Alelle	MAF
4	rs13120400	C	T	0.2673
16	rs35592	C	T	0.2277
16	rs2238476	A	G	0.0495
16	rs246240	G	A	0.1485
23	rs3761548	G	T	0.4803
Chr: Chromosome; MAF: Minor allele frequency				
