# Supplementary material for: ABCC1, ABCG2 and FOXP3: Predictive Biomarkers of Toxicity from Methotrexate Treatment in Patients Diagnosed with Moderate-to-Severe Psoriasis
Source: Biomedicines. 2023 Sep 19;11(9):2567. doi: 10.3390/biomedicines11092567 (PMC10526923; doi:10.3390/biomedicines11092567)
Supplement: Supplementary file 1 [file biomedicines-11-02567-s001.zip › Table S15. SNP and overall toxicity.pdf]

Table S15. Single nucleotide polymorphisms and overall toxicity.

Gene	SNP	Genotype	N	Overall toxicity		χ^2	p-value	OR	$IC_{95\%}$
				NO N (%)	YES (Grado 1-4) N (%)				
ABCC1	rs246240	AA	74	23 (31.1)	51 (68.9)	-	1*	-	-
		AG	24	8(33.3)	16(66.7)				
		GG	3	1(33.3)	2(66.7)				
		A	98	31(31.6)	67(68.4)	-	1*	-	-
		G	27	9(33.3)	18(66.7)	0.046	0.829	-	-
	rs35592	CC	3	1(33.3)	2(66.7)	-	0.922*	-	-
		CT	40	12(30.0)	28(70.0)				
		TT	58	19(32.8)	39(67.2)				
		C	43	13(30.2)	30(69.8)	0.073	0.787	**-**	**-**
		T	98	31(31.6)	67(68.4)	-	1*	**-**	**-**
	rs2238476	GG	91	29(31.9)	62(68.1)	-	1*	**-**	**-**
		AG	10	3(30.0)	7(70.0)				
		A	10	3 (30.0)	7 (70.0)	-	1*	-	-
ABCG2	rs13120400	TT	53	14(26.4)	39(73.6)	-	**0.023***	**13.93**	**2.02-279.52**
		CT	42	13(31.0)	29(69.0)			**11.15**	**1.59-225.34**
		CC	6	5(83.3)	1(16.7)			**1**	**-**
		T	95	27(28.4)	68(71.6)	-	**0.012***	**12.59**	**1.92-247.28**
		C	48	18(37.5)	30(62.5)	1.429	0.232	-	-
FOXP3	rs3761548	GG	32	10 (31.2)	22 (68.8)	1.391	0.499	-	-
		GT	29	7 (24.1)	22 (75.9)				
		TT	40	15 (37.5)	25 (62.5)				
		G	61	17 (27.9)	44 (72.1)	1.035	0.309	-	-
		T	69	22 (31.9)	47 (68.1)	0.004	0.949	-	-

*p-value for Fisher's test.
