# Supplementary material for: ABCC1, ABCG2 and FOXP3: Predictive Biomarkers of Toxicity from Methotrexate Treatment in Patients Diagnosed with Moderate-to-Severe Psoriasis
Source: Biomedicines. 2023 Sep 19;11(9):2567. doi: 10.3390/biomedicines11092567 (PMC10526923; doi:10.3390/biomedicines11092567)
Supplement: Supplementary file 1 [file biomedicines-11-02567-s001.zip › Table S16. SNP and more than 1 AE.pdf]

Table S16. Single nucleotide polymorphisms and more than one adverse event.

Gene	SNP	Genotype	N	More than 1 adverse event		χ^2	p-value	OR	$IC_{95\%}$
				NO N (%)	YES (Grado 1-4) N (%)				
ABCC1	rs246240	**AA**	74	49(66.2)	25(33.8)	-	0.831*	-	-
		**AG**	24	14(58.3)	10(41.7)				
		**GG**	3	2(66.7)	1(33.3)				
		**A**	98	63(64.3)	35(35.7)	-	1*	-	-
		**G**	27	16(59.3)	11(40.7)	0.417	0.518	-	-
	rs35592	**CC**	3	2(66.7)	1(33.3)	-	0.924*	-	-
		**CT**	40	25(62.5)	15(37.5)				
		**TT**	58	38(65.5)	20(34.5)				
		**C**	43	27(62.8)	16(37.2)	0.080	0.777	**-**	**-**
		**T**	98	63(64.3)	35(35.7)		1*	**-**	**-**
	rs2238476	**GG**	91	61(67.0)	30(33.0)	-	0.160*	**-**	**-**
		**AG**	10	4(40.0)	6(60.0)				
		**A**	10	4 (40.0)	6(60.0)	-	0.160*	-	-
ABCG2	rs13120400	**TT**	53	32(60.4)	21(39.6)	-	0.529*	-	-
		**CT**	42	28(66.7)	14(33.3)				
		**CC**	6	5(83.3)	1(16.7)				
		**T**	95	60(63.2)	35(36.8)	-	0.417*	-	-
		**C**	48	33(68.8)	15(31.2)	-	0.412*	-	-
FOXP3	rs3761548	**GG**	32	23 (71.9)	9 (28.1)	6.767	**0.034**	**1.03**	**0.36-2.91**
		**GT**	29	13 (44.8)	16 (55.2)			**3.24**	**1.20-9.15**
		**TT**	40	29 (72.5)	11 (27.5)			**1**	**-**
		**G**	61	36 (59.0)	25 (41.0)	1.915	0.166	-	-
		**T**	69	42 (60.9)	27 (39.1)	1.154	0.283	-	-

*p-value for test Fisher's
