# Supplementary material for: ABCC1, ABCG2 and FOXP3: Predictive Biomarkers of Toxicity from Methotrexate Treatment in Patients Diagnosed with Moderate-to-Severe Psoriasis
Source: Biomedicines. 2023 Sep 19;11(9):2567. doi: 10.3390/biomedicines11092567 (PMC10526923; doi:10.3390/biomedicines11092567)
Supplement: Supplementary file 1 [file biomedicines-11-02567-s001.zip › Table S17. SNP and more than 2 adverse events.pdf]

Table S17. Single nucleotide polymorphisms and more than two adverse events.

Gene	SNP	Genotype	N	More than 2 adverse events		X^2	p-value	OR	$IC_{95\%}$
				NO N (%)	YES (Grado 1-4) N (%)				
ABCC1	rs246240	AA	74	61(82.4)	13(17.6)	-	0.599*	-	-
		AG	24	22(91.7)	2(8.3)				
		GG	3	3(100.0)	0(0.0)				
		A	98	83(84.7)	15(15.3)	-	1*	-	-
		G	27	25(92.6)	2(7.4)	-	0.343*	-	-
	rs35592	CC	3	3(100.0)	0(0.0)	-	0.405*	-	-
		CT	40	36(90.0)	4(10.0)				
		TT	58	47(81.0)	11(19.0)				
		C	43	39(90.7)	4(9.3)	1.8233	0.176	-	-
		T	98	83(84.7)	15(15.3)	-	1*	-	-
	rs2238476	GG	91	80(87.9)	11(12.1)	-	**0.039***	**1**	-
		AG	10	6(60.0)	4(40.0)			**4.85**	**1.09-19.89**
		A	10	6(60.0)	4(40.0)	-	**0.039***	4.85	**1.09-19.89**
ABCG2	rs13120400	TT	53	46(86.8)	7(13.2)	-	0.906*	-	-
		CT	42	35(83.3)	7(16.7)				
		CC	6	5(83.3)	1(16.7)				
		T	95	81(85.3)	14(14.7)	0.017	0.897	-	-
		C	48	40(83.3)	8(16.7)	0.238	0.625	-	-
FOXP3	rs3761548	GG	32	29 (90.6)	3 (9.4)	-	**0.021***	**1.28**	**0.22-7.34**
		GT	29	20 (69.0)	9 (31.0)			**5.55**	**1.47-27.20**
		TT	40	37 (92.5)	3 (7.5)			**1**	-
		G	61	49 (80.3)	12 (19.7)	2.830	0.092	-	-
		T	69	57 (82.6)	12 (17.4)	-	0.377*	-	-

* p-value by Fisher's test.
