# Supplementary material for: ABCC1, ABCG2 and FOXP3: Predictive Biomarkers of Toxicity from Methotrexate Treatment in Patients Diagnosed with Moderate-to-Severe Psoriasis
Source: Biomedicines. 2023 Sep 19;11(9):2567. doi: 10.3390/biomedicines11092567 (PMC10526923; doi:10.3390/biomedicines11092567)
Supplement: Supplementary file 1 [file biomedicines-11-02567-s001.zip › Table S18. SNP and hepatotoxicity.pdf]

Table S18. Single nucleotide polymorphisms and hepatotoxicity.

Gene	SNP	Genotype	N	Hepatotoxicity		X^2	p-value	OR	$IC_{95\%}$
				NO N (%)	YES (Grade 1-4) N (%)				
ABCC1	rs246240	AA	74	46(62.2)	28(37.8)	-	0.945*	-	-
		AG	24	16(66.7)	8(33.3)				
		GG	3	2(66.7)	1(33.3)				
		A	98	62(63.3)	36(36.7)	-	1*	-	-
		G	27	18(66.7)	9(33.3)	0.173	0.678	-	-
	rs35592	CC	3	3(100.0)	0(0.0)	-	0.369*		
		CT	40	23(57.5)	17(42.5)				
		TT	58	38(65.5)	20(34.5)				
		C	43	26(60.5)	17(39.5)	0.272	0.602	-	-
		T	98	61(62.2)	37(37.8)	-	0.297*	-	-
	rs2238476	GG	91	56(61.5)	35(38.5)	-	0.318*	-	-
		AG	10	8(80.0)	2(20.0)				
		A	10	8(80.0)	2(20.0)	-	0.318*	-	-
ABCG2	rs13120400	TT	53	29(54.7)	24(45.3)	5.760	0.056	-	-
		CT	42	29(69.0)	13(31.0)				
		CC	6	6(100.0)	0(0.0)				
		T	95	58(61.1)	37(38.9)	-	0.083*	-	-
		C	48	35(72.9)	13(27.1)	3.594	**0.058**	**0.44**	**0.19-1.02**
FOXP3	rs3761548	GG	32	20 (62.5)	12 (37.5)	0.586	0.746	-	-
		GT	29	17 (58.6)	12 (41.4)				
		TT	40	27 (67.5)	13 (32.5)				
		G	61	37 (60.7)	24 (39.3)	0.488	0.485	-	-
		T	69	44 (63.8)	25 (36.2)	0.015	0.902	-	-

*p-value by Fisher's test.
