# Supplementary material for: ABCC1, ABCG2 and FOXP3: Predictive Biomarkers of Toxicity from Methotrexate Treatment in Patients Diagnosed with Moderate-to-Severe Psoriasis
Source: Biomedicines. 2023 Sep 19;11(9):2567. doi: 10.3390/biomedicines11092567 (PMC10526923; doi:10.3390/biomedicines11092567)
Supplement: Supplementary file 1 [file biomedicines-11-02567-s001.zip › Table S19. SNP and gastrointestinal toxicity.pdf]

Table S19. Single nucleotide polymorphisms and gastrointestinal toxicity.

Gene	SNP	Genotype	N	Gastrointestinal toxicity		X^2	p-value	OR	$IC_{95\%}$
				NO N (%)	YES (Grado 1-4) N (%)				
ABCC1	rs246240	AA	74	54(73.0)	20(27.0)	-	0.825*	-	-
		AG	24	16(66.7)	8(33.3)				
		GG	3	2(66.7)	1(33.3)				
		A	98	70(71.4)	28(28.6)	-	1*	-	-
		G	27	18(66.7)	9(33.3)	0.384	0.535	-	-
	rs35592	CC	3	2(66.7)	1(33.3)	-	0.492*	-	-
		CT	40	31(77.5)	9(22.5)				
		TT	58	39(67.2)	19(32.8)				
		C	43	33(76.7)	10(23.3)	1.089	0.297	-	-
		T	98	70(71.4)	28(28.6)	-	1*	-	-
	rs2238476	GG	91	67(73.6)	24(26.4)	-	0.145*	-	-
		AG	10	5(50.0)	5(50.0)				
		A	10	5(50.0)	5(50.0)	2.457	0.117	-	-
ABCG2	rs13120400	TT	53	39(73.6)	14(26.4)	3.559	0.169	-	-
		CT	42	27(64.3)	15(35.7)				
		CC	6	6(100.0)	0(0.0)				
		T	95	66(69.5)	29(30.5)	-	0.173*	-	-
		C	48	33(68.8)	15(31.2)	0.288	0.592	-	-
FOXP3	rs3761548	GG	32	24 (75.0)	8 (25.0)	1.689	0.429	-	-
		GT	29	18 (62.1)	11 (37.9)				
		TT	40	30 (75.0)	10 (25.0)				
		G	61	42 (68.9)	19 (31.1)	0.446	0.504	-	-
		T	69	48 (69.6)	21 (30.4)	0.315	0.574	-	-

*P-value by Fisher's test.
