# Supplementary material for: ABCC1, ABCG2 and FOXP3: Predictive Biomarkers of Toxicity from Methotrexate Treatment in Patients Diagnosed with Moderate-to-Severe Psoriasis
Source: Biomedicines. 2023 Sep 19;11(9):2567. doi: 10.3390/biomedicines11092567 (PMC10526923; doi:10.3390/biomedicines11092567)
Supplement: Supplementary file 1 [file biomedicines-11-02567-s001.zip › Table S2. Clinical variables and more than 1 adverse event.pdf]

Table S2. Clinical variables and more than 1 adverse event

Characteristics	N	More than 1 adverse event		X^2	p-valor	OR	$IC_{95\%}$
		**NO N (%)**	**YES (Grade 1-4) N (%)**				
**Gender**	101						
Female	52	27 (51.9)	25 (48.1)	7.223	**0.007**	**3.20**	**1.37-7.82**
Male	49	38 (77.6)	11 (22.4)			**1**	**-**
**Age diagnosis PS**	101	28.5 (19.3-45.8)	25.6 (13.6-41.7)	-	0.326	-	-
**Family History of Ps**	101						
Yes	52	36 (69.2)	16 (30.8)	1.11	0.292	-	-
No	49	29 (59.2)	20 (40.8)				
**Smoking**	101						
Smoker	31	25 (80.6)	6 (19.4)	7.843	**0.019**	**1**	**-**
Non-smoking	49	31 (63.3)	18 (36.7)			**2.42**	**0.87-7.51**
Former Smoker	21	9 (42.9)	12 (57.1)			**5.56**	**1.67-20.48**
**Alcoholic drinking**	101						
Drinker	38	29 (76.3)	9 (23.7)	-	0.094*	-	-
Non-drinker	61	35 (57.4)	26 (42.6)				
Former Drinker	2	1 (50.0)	1 (50.0)				
**Type of Psoriasis**	101						
Plaque	74	48(64.9)	26(35.1)	-	0.949*	-	-
Pustular	5	3(60.0)	2(40.0)				
Inverse	1	1(100.0)	0(0.0)				
Guttate	5	2(40.0)	3(60.0)				
Plaque and guttate	12	8(66.7)	4(33.3)				
Plaque and inverse	2	1(50.0)	1(50.0)				
Plaque and pustular	1	1(100.0)	0(0.0)				
Plaque, guttate and inverse	1	1(100.0)	0(0.0)				
**Localization**							
Trunk and limbs	101						
Yes	93	59(63.4)	34(36.6)	-	0.708*	-	-
No	8	6(75.0)	2(25.0)				
Scalp and face	101						
Yes	77	48(62.3)	29(37.7)	0.576	0.448	-	-
No	24	17(70.8)	7(29.2)				
Nails	101						
Yes	58	33(56.9)	25(43.1)	3.305	0.069	**-**	**-**
No	43	32(74.4)	11(25.6)				
Palmoplantar	101						
Yes	19	14(73.7)	5(26.3)	0.887	0.346	-	-
No	82	51(62.2)	31(37.8)				
Flexures	101						
Yes	28	14 (50.0)	14 (50.0)	3.481	**0.062**	**2.31**	**0.94-5.72**
No	73	51 (69.9)	22 (30.1)			**1**	**-**
**Development of PSA**	101						
Yes	31	14(45.2)	17(54.8)	7.185	**0.007**	**3.26**	**1.36-8.01**
No	70	51(72.9)	19(27.1)			**1**	**-**
**Comorbidities**	101						

Yes	57	33 (57.9)	24 (42.1)	2.381	0.123	-	-
No	44	32 (72.7)	12 (27.3)				
**Age of onset of MTX**	101	46.52±15.53	43.94±13.42	-	0.385	-	-
**MTX therapy duration (months)**	101	12.0 (5.0-24.0)	17.0 (4.8-42.8)	-	**0.063**	**1.02**	**1.00-1.04**
**MTX Administration**	101						
Oral	47	40 (85.1)	7 (14.9)	22.026	**<0.001**	**1**	**-**
Subcutaneous	30	18 (60.0)	12 (40.0)			**3.81**	**1.32-11.81**
Both	24	7 (29.2)	17 (70.8)			**13.88**	**4.45-49.21**
**Type of MTX therapy**	101						
Monotherapy	93	60 (64.5)	33 (35.5)	0.013	0.909	-	-
Combination Therapy	8	5 (62.5)	3 (37.5)				
**Maximum MTX dose (mg/week)**	101	15.0 (10.0-15.0)	12.5 (10.0-15.0)	-	0.865	-	-
**Therapeutic adherence**	101						
Adherent	70	49 (70.0)	21 (30.0)	-	0.107*	-	-
Intentional non-adherent	30	15 (50.0)	15 (50.0)				
Unintentional non-adherent	1	1 (100.0)	0 (0.0)				

*p-value for Fisher's test
PS: psoriasis; PSA: psoriatic arthritis
