# Supplementary material for: ABCC1, ABCG2 and FOXP3: Predictive Biomarkers of Toxicity from Methotrexate Treatment in Patients Diagnosed with Moderate-to-Severe Psoriasis
Source: Biomedicines. 2023 Sep 19;11(9):2567. doi: 10.3390/biomedicines11092567 (PMC10526923; doi:10.3390/biomedicines11092567)
Supplement: Supplementary file 1 [file biomedicines-11-02567-s001.zip › Table S20. SNP and asthenia.pdf]

Tabla S20. Single nucleotide polymorphisms and asthenia

Gen	SNP	Genotype	N	Asthenia		X^2	p-value	OR	$IC_{95\%}$
				**NO N (%)**	**YES (Grade 1-4) N (%)**				
ABCC1	**rs246240**	**AA**	74	53(71.6)	21(28.4)	-	1*	-	-
		**AG**	24	18(75.0)	6(25.0)				
		**GG**	3	2(66.7)	1(33.3)				
		**A**	98	71(72.4)	27(27.6)	-	1*	-	-
		**G**	27	20(74.1)	7(25.9)	0.059	0.808	-	-
	**rs35592**	**CC**	3	2(66.7)	1(33.3)	-	1*	-	-
		**CT**	40	29(72.5)	11(27.5)				
		**TT**	58	42(72.4)	16(27.6)				
		**C**	43	31(72.1)	12(27.9)	0.001	0.972	**-**	**-**
		**T**	98	71(72.4)	27(27.6)	-	1*	**-**	**-**
	**rs2238476**	**GG**	91	69(75.8)	22(24.2)	-	**0.025***	**1**	**-**
		**AG**	10	4(40.0)	6(60.0)			**4.70**	**1.23-19.87**
		**A**	10	4(40.0)	6(60.0)	-	**0.026***	**4.70**	**1.23-19.87**
ABCG2	**rs13120400**	**TT**	53	39(73.6)	14(26.4)	-	0.769*	-	-
		**CT**	42	29(69.0)	13(31.0)				
		**CC**	6	5(83.3)	1(16.7)				
		**T**	95	68(71.6)	27(28.4)	-	1*	-	-
		**C**	48	34(70.8)	14(29.2)	0.095	0.758	-	-
FOXP3	**rs3761548**	**GG**	32	25 (78.1)	7 (21.9)	5.971	**0.051**	**1.12**	**0.35-3.53**
		**GT**	29	16 (55.2)	13 (44.8)			**3.25**	**1.14-9.79**
		**TT**	40	32 (80.0)	8 (20.0)			**1**	**-**
		**G**	61	41 (67.2)	20 (32.8)	1.971	0.160	-	-
		**T**	69	48 (69.6)	21 (30.4)	0.799	0.371	-	-

*p-value por el test de Fisher's
