# Supplementary material for: ABCC1, ABCG2 and FOXP3: Predictive Biomarkers of Toxicity from Methotrexate Treatment in Patients Diagnosed with Moderate-to-Severe Psoriasis
Source: Biomedicines. 2023 Sep 19;11(9):2567. doi: 10.3390/biomedicines11092567 (PMC10526923; doi:10.3390/biomedicines11092567)
Supplement: Supplementary file 1 [file biomedicines-11-02567-s001.zip › Table S21. SNP and nephrotoxicity.pdf]

Table S21. Single nucleotide polymorphisms and nephrotoxicity.

Gen	SNP	Genotype	N	Nephrotoxicity		X^2	p-value	OR	$IC_{95\%}$
				NO<br>N (%)	YES<br>(Grade 1-4)<br>N (%)				
ABCC1	rs246240	AA	74	73(98.6)	1(1.4)	-	1*	-	-
		AG	24	24(100.0)	0(0.0)				
		GG	3	3(100.0)	0(0.0)				
		A	98	97(99.0)	1(1.0)	-	1*	-	-
		G	27	27(100.0)	0(0.0)	-	1*	-	-
	rs35592	CC	3	3(100.0)	0(0.0)	-	1*	-	-
		CT	40	40(100.0)	0(0.0)				
		TT	58	57(98.3)	1(1.7)				
		C	43	43(100.0)	0(0.0)	-	1*	-	-
		T	98	97(99.0)	1(1.0)	-	1*	-	-
	rs2238476	GG	91	91(100.0)	0(0.0)	-	**0.099***	-	-
		AG	10	9(90.0)	1(10.0)			-	-
		A	10	9(90.0)	1(10.0)	-	**0.099***	-	-
ABCG2	rs13120400	TT	53	53(100.0)	0(0.0)	-	0.475*	-	-
		CT	42	41(97.6)	1(2.4)				
		CC	6	6(100.0)	0(0.0)				
		T	95	94(98.9)	1(1.1)	-	1*	-	-
		C	48	47(97.9)	1(2.1)	-	0.475*	-	-
FOXP3	rs3761548	GG	40	40 (100.0)	0 (0.0)	-	0.287*	-	-
		GT	29	28 (96.6)	1 (3.4)				
		TT	32	32 (100.0)	0 (0.0)				
		G	61	60 (98.4)	1 (1.6)	-	1*	-	-
		T	69	68 (98.6)	1 (1.4)	-	1*	-	-

*p-value by Fisher's test.
