# Supplementary material for: ABCC1, ABCG2 and FOXP3: Predictive Biomarkers of Toxicity from Methotrexate Treatment in Patients Diagnosed with Moderate-to-Severe Psoriasis
Source: Biomedicines. 2023 Sep 19;11(9):2567. doi: 10.3390/biomedicines11092567 (PMC10526923; doi:10.3390/biomedicines11092567)
Supplement: Supplementary file 1 [file biomedicines-11-02567-s001.zip › Table S22. SNP and Neurotoxicity.pdf]

Table S22. Single nucleotide polymorphisms and neurotoxicity.

Gene	SNP	Genotype	N	Neurotoxicity		X^2	p-value	OR	$IC_{95\%}$
				NO N (%)	YES (Grade 1-4) N (%)				
ABCC1	rs246240	AA	74	66(89.2)	8(10.8)	-	0.583*	-	-
		AG	24	23(95.8)	1(4.2)				
		GG	3	3(100.0)	0(0.0)				
		A	98	89(90.8)	9(9.2)	-	1*	-	-
		G	27	26(96.3)	1(3.7)	-	0.438*	-	-
	rs35592	CC	3	3(100.0)	0(0.0)	-	1*	-	-
		CT	40	36(90.0)	4(10.0)				
		TT	58	53(91.4)	5(8.6)6				
		C	43	39(90.7)	4(9.3)	-	1*	-	-
		T	98	89(90.8)	9(9.2)	-	1*		--
	rs2238476	GG	91	84(92.3)	7(7.7)	-	0.218*	-	-
		AG	10	8(80.0)	2(20.0)				
		A	10	8(80.0)	2(20.0)	-	0.218*	-	-
ABCG2	rs13120400	TT	53	48(90.6)	5(9.4)	-	1*	-	-
		CT	42	38(90.5)	4(9.5)			-	-
		CC	6	6(100.0)	0(0.0)			-	-
		T	95	86(90.5)	9(9.5)	-	1*	-	-
		C	48	44(91.7)	4(8.3)	-	1*	-	-
FOXP3	rs3761548	GG	40	37 (92.5)	3 (7.5)	-	0.614*	-	-
		GT	29	25 (86.2)	4 (13.8)			-	-
		TT	32	30 (93.8)	2 (6.2)			-	-
		G	61	55 (90.2)	6 (9.8)	-	1*	-	-
		T	69	62 (89.9)	7 (10.1)	-	0.715*	-	-

*p-value by Fisher's test.
