# Supplementary material for: ABCC1, ABCG2 and FOXP3: Predictive Biomarkers of Toxicity from Methotrexate Treatment in Patients Diagnosed with Moderate-to-Severe Psoriasis
Source: Biomedicines. 2023 Sep 19;11(9):2567. doi: 10.3390/biomedicines11092567 (PMC10526923; doi:10.3390/biomedicines11092567)
Supplement: Supplementary file 1 [file biomedicines-11-02567-s001.zip › Table S23. SNP and cutaneous toxicity.pdf]

Table S23. Single nucleotide polymorphisms and cutaneous toxicity.

Gene	SNP	Genotype	N	Cutaneous toxicity		X^2	p-value	OR	$IC_{95\%}$
				NO N (%)	YES (Grade 1-4) N (%)				
ABCC1	rs246240	AA	74	70(94.6)	4(5.4)	-	0.165*	-	-
		AG	24	20(83.3)	4(16.7)				
		GG	3	3(100.0)	0(0.0)				
		A	98	90(91.8)	8(8.2)	-	1*	-	-
		G	27	23(85.2)	4(14.8)	-	0.205*	-	-
	rs35592	CC	3	2(66.7)	1(33.3)	-	0.153*	-	-
		CT	40	36(90.0)	4(10.0)				
		TT	58	55(94.8)	3(5.2)				
		C	43	38(88.4)	5(11.6)	-	0.280*	-	-
		T	98	91(92.9)	7(7.1)	-	0.221*	-	-
	rs2238476	GG	91	83(91.2)	8(8.8)	-	1*	-	-
		AG	10	10(100.0)	0(0.0)				
		A	10	10(100.0)	0(0.0)	-	1*	-	-
ABCG2	rs13120400	TT	53	47(88.7)	6(11.3)	-	0.125*	-	-
		CT	42	41(97.6)	1(2.4)			-	-
		CC	6	5(83.3)	1(16.7)			-	-
		T	95	88(92.6)	7(7.4)	-	0.399*	-	-
		C	48	46(95.8)	2(4.2)	-	0.274*	-	-
FOXP3	rs3761548	GG	40	36 (90.0)	4 (10.0)	-	0.902*	-	-
		GT	29	27 (93.1)	2 (6.9)			-	-
		TT	32	30 (93.8)	2 (6.2)			-	-
		G	61	57 (93.4)	4 (6.6)	-	0.709*	-	-
		T	69	63 (61.3)	6 (8.7)	-	1*	-	-

*p-value by Fisher's test.
