# Supplementary material for: ABCC1, ABCG2 and FOXP3: Predictive Biomarkers of Toxicity from Methotrexate Treatment in Patients Diagnosed with Moderate-to-Severe Psoriasis
Source: Biomedicines. 2023 Sep 19;11(9):2567. doi: 10.3390/biomedicines11092567 (PMC10526923; doi:10.3390/biomedicines11092567)
Supplement: Supplementary file 1 [file biomedicines-11-02567-s001.zip › Table S24. SNP and hematological toxicity.pdf]

Table S24. Single nucleotide polymorphisms and hematological toxicity.

Gene	SNP	Genotype	N	Hematological toxicity		χ^2	p-value	OR	$IC_{95\%}$
				NO N (%)	YES (Grade 1-4) N (%)				
ABCC1	rs246240	AA	74	72(97.3)	2(2.7)	-	1*	-	-
		AG	24	23(95.8)	1(4.2)				
		GG	3	3(100.0)	0(0.0)				
		A	98	95(96.9)	3(3.1)	-	1*	-	-
		G	27	26(96.3)	1(3.7)	-	1*	-	-
	rs35592	CC	3	3(100.0)	0(0.0)	-	0.6032*	-	-
		CT	40	38(95.0)	2(5.0)				
		TT	58	57(98.3)	1(1.7)				
		C	43	41(95.3)	2(4.7)	-	0.5735*	-	-
		T	98	95(96.9)	3(3.1)	-	1*	-	-
	rs2238476	GG	91	88(96.7)	3(3.3)	-	1*	-	-
		AG	10	10(100.0)	0(0.0)				
		A	10	10(100.0)	0(0.0)	-	1*	-	-
ABCG2	rs13120400	TT	53	51(96.2)	2(3.8)	-	1*	-	-
		CT	42	41(97.6)	1(2.4)			-	-
		CC	6	6(100.0)	0(0.0)			-	-
		T	95	92(96.8)	3(3.2)	-	1*	-	-
		C	48	47(97.9)	1(2.1)	-	1*	-	-
FOXP3	rs3761548	GG	32	31 (96.9)	1 (3.1)	-	0.189*	-	-
		GT	29	27 (93.1)	2 (6.9)			-	-
		TT	40	40 (100.0)	0 (0.0)			-	-
		G	61	58 (95.1)	3 (4.9)	-	0.275*	-	-
		T	69	67 (97.1)	2 (2.9)	-	1*	-	-

*p-value by Fisher's test.
