# Supplementary material for: ABCC1, ABCG2 and FOXP3: Predictive Biomarkers of Toxicity from Methotrexate Treatment in Patients Diagnosed with Moderate-to-Severe Psoriasis
Source: Biomedicines. 2023 Sep 19;11(9):2567. doi: 10.3390/biomedicines11092567 (PMC10526923; doi:10.3390/biomedicines11092567)
Supplement: Supplementary file 1 [file biomedicines-11-02567-s001.zip › Table S25. Single nucleotide polymorphisms and infections.pdf]

Table S25. Single nucleotide polymorphisms and infections.

Gene	SNP	Genotype	N	Infections		X^2	p-value	OR	$IC_{95\%}$
				NO N (%)	YES (Grado 1-4) N (%)				
ABCC1	**rs246240**	**AA**	74	69(93.2)	5(6.8)	-	1*	-	-
		**AG**	24	23(95.8)	1(4.2)				
		**GG**	3	3(100.0)	0(0.0)				
		**A**	98	92(93.9)	6(6.1)	-	1*	-	-
		**G**	27	26(96.3)	1(3.7)	-	1*	-	-
	**rs35592**	**CC**	3	3(100.0)	0(0.0)	-	0.4983*	-	-
		**CT**	40	39(97.5)	1(2.5)				
		**TT**	58	53(91.4)	5(8.6)				
		**C**	43	42(97.7)	1(2.3)	-	0.2363*	-	-
		**T**	98	92(93.9)	6(6.1)	-	1*	-	-
	**rs2238476**	**GG**	91	86(94.5)	5(5.5)	-	0.474*	-	-
		**AG**	10	9(90.0)	1(10.0)				
		**A**	10	9(90.0)	1(10.0)	-	0.474*	-	-
ABCG2	**rs13120400**	**TT**	53	51(96.2)	2(3.8)	-	0.277*	-	-
		**CT**	42	39(92.9)	3(7.1)			-	-
		**CC**	6	5(83.3)	1(16.7)			-	-
		**T**	95	90(94.7)	5(5.3)		0.3142*	-	-
		**C**	48	44(91.7)	4(8.3)		0.4197*	-	-
FOXP3	**rs3761548**	**GG**	32	30 (93.8)	2 (6.2)	-	1*	-	-
		**GT**	29	27 (93.1)	2 (6.9)			-	-
		**TT**	40	38 (95.0)	2 (5.0)			-	-
		**G**	61	57 (93.4)	4 (6.6)	-	1*	-	-
		**T**	69	65 (94.2)	4 (5.8)	-	1*	-	-

*p-value by Fisher's test.
