# Supplementary material for: ABCC1, ABCG2 and FOXP3: Predictive Biomarkers of Toxicity from Methotrexate Treatment in Patients Diagnosed with Moderate-to-Severe Psoriasis
Source: Biomedicines. 2023 Sep 19;11(9):2567. doi: 10.3390/biomedicines11092567 (PMC10526923; doi:10.3390/biomedicines11092567)
Supplement: Supplementary file 1 [file biomedicines-11-02567-s001.zip › Table S3. Clinical variables and more than 2 adverse events.pdf]

Table S3. Clinical variables and more than 2 adverse events.

Characteristics	N	More than 2 adverse events		χ^2	p-value	OR	$IC_{95\%}$
		NO N (%)	YES (Grade 1-4) N (%)				
**Gender**	101						
Female	52	40(76.9)	12(23.1)	5.735	**0.017**	**4.60**	**1.35-21.24**
Male	49	46(93.9)	3(6.1)			**1**	**-**
**Age diagnosis PS**	101	27.1 (19.0-45.2)	28.8 (15.13-40.96)	-	0.465	-	-
**Family History of Ps**	101						
Yes	52	42 (80.8)	10 (19.2)	1.626	0.202	-	-
No	49	44 (89.9)	5 (10.2)				
**Smoking**	101						
Smoker	31	29 (93.5)	2 (6.5)	-	0.197*	**-**	**-**
Non-smoking	49	41 (83.7)	8 (16.3)				
Former Smoker	21	16 (76.2)	5 (23.8)				
**Alcoholic drinking**	101						
Drinker	38	35 (92.1)	3 (7.9)	-	0.273*	-	-
Non-drinker	61	49 (80.3)	12 (19.7)				
Former Drinker	2	2 (100.0)	0 (0.0)				
**Type of Psoriasis**	101						
Plaque	74	65(87.8)	9(12.2)	-	0.166*	-	-
Pustular	5	5(100.0)	0(0.0)				
Inverse	1	1(100.0)	0(0.0)				
Guttate	5	2(40.0)	3(60.0)				
Plaque and guttate	12	9(75.0)	3(25.0)				
Plaque and inverse	2	2(100.0)	0(0.0)				
Plaque and pustular	1	1(100.0)	0(0.0)				
Plaque, guttate and inverse	1	1(100.0)	0(0.0)				
**Localization**							
Trunk and limbs	101						
Yes	93	79(84.9)	14(15.1)	-	1*	-	-
No	8	7(87.5)	1(12.5)				
Scalp and face	101						
Yes	77	63(81.8)	14(18.2)	-	0.111 *	-	-
No	24	23(95.8)	1(4.2)				
Nails	101						
Yes	58	47(81.0)	11(19.0)	1.82	0.177	**-**	**-**
No	43	39(90.7)	4(9.3)				
Palmoplantar	101						
Yes	19	18(94.7)	1(5.3)	-	0.291*	-	-
No	82	68(82.9)	14(17.1)				
Flexures	101						
Yes	73	64 (87.7)	9 (12.3)	-	0.347*	**-**	**-**
No	28	22 (78.6)	6 (21.4)				
**Development of PSA**	101						
Yes	31	24(77.4)	7(22.6)	-	0.223*	**-**	**-**
No	70	62(88.6)	8(11.4)				
**Comorbidities**	101						
Yes	57	50(87.7)	7(12.3)	0.684	0.408	-	-
No	44	36(81.8)	8(18.2)				
**Age of onset of MTX**	101	45.86±15.41	44.13±10.93	-	0.603	-	-

**MTX therapy duration (months)**	101	14.5 (5.0-32.5)	15.0 (4.5-33.5)	-	0.563	**-**	**-**
**MTX Administration**	101						
Oral	47	44 (93.6)	3 (6.4)	-	**0.038***	**1**	**-**
Subcutaneous	30	25 (83.3)	5 (16.7)			**2.93**	**0.66-15.29**
Both	24	17 (70.8)	7 (29.2)			**6.04**	**1.49-30.62**
**Type of MTX therapy**	101						
Monotherapy	93	80 (86.0)	13 (14.0)	-	0.339*	-	-
Combination Therapy	8	6 (75.0)	2 (25.0)				
**Maximum MTX dose (mg/week)**	101	12.5 (10.0-15.0)	15.0 (10.0-15.0)	-	0.711	-	-
**Therapeutic adherence**	101						
Adherent	70	61 (87.1)	9 (12.9)	-	0.466*	-	-
Intentional non-adherent	30	24 (80.0)	6 (20.0)				
Unintentional non-adherent	1	1 (100.0)	0 (0.0)				

*p-value for the Fisher's test. PS: psoriasis; PSA: psoriatic arthritis
