# Supplementary material for: ABCC1, ABCG2 and FOXP3: Predictive Biomarkers of Toxicity from Methotrexate Treatment in Patients Diagnosed with Moderate-to-Severe Psoriasis
Source: Biomedicines. 2023 Sep 19;11(9):2567. doi: 10.3390/biomedicines11092567 (PMC10526923; doi:10.3390/biomedicines11092567)
Supplement: Supplementary file 1 [file biomedicines-11-02567-s001.zip › Table S4. Clinical variables and hepatotoxicity.pdf]

Table S4. Clinical variables and hepatotoxicity

Characteristics	N	Hepatotoxicity		X^2	p-valor	OR	$IC_{95\%}$
		NO N (%)	YES (Grado 1-4) N (%)				
**Gender**	101						
Female	52	35(67.3)	17(32.7)	0.717	0.397	-	-
Male	49	29(59.2)	20(40.8)				
**Age diagnosis PS**	101	29.2 (17.6-46.4)	26.7 (19.1-39.1)	-	0.613	-	-
**Family History of Ps**	101						
Yes	52	34 (65.4)	18 (34.6)	0.188	0.665	-	-
No	49	30 (61.2)	19 (38.8)				
**Smoking**	101						
Smoker	31	24 (77.4)	7 (22.6)	4.098	0.129	-	-
Non-smoking	49	27 (55.1)	22 (44.9)				
Former Smoker	21	13 (61.9)	8 (38.1)				
**Alcoholic drinking**	101						
Drinker	38	22 (57.9)	16 (42.1)	-	0.474*	-	-
Non-drinker	61	40 (65.6)	21 (34.4)				
Former Drinker	2	2 (100.0)	0 (0.0)				
**Type of Psoriasis**	101						
Plaque	74	47(63.5)	27(36.5)	-	0.102*	-	-
Pustular	5	4(80.0)	1(20.0)				
Inverse	1	0(0.0)	1(100.0)				
Guttate	5	5(100.0)	0(0.0)				
Plaque and guttate	12	7(58.3)	5(41.7)				
Plaque and inverse	2	0(0.0)	2(100.0)				
Plaque and pustular	1	1(100.0)	0(0.0)				
Plaque, guttate and inverse	1	0(0.0)	1(100.0)				
**Localization**							
Trunk and limbs	101						
Yes	93	58(62.4)	35(37.6)	0.507	0.477	-	-
No	8	6(75.0)	2(25.0)				
Scalp and face	101						
Yes	77	47(61.0)	30(39.0)	0.756	0.385	-	-
No	24	17(70.8)	7(29.2)				
Nails	101						
Yes	58	34(58.6)	24(41.4)	1.322	0.250	-	-
No	43	30(69.8)	13(30.2)				
Palmoplantar	101						
Yes	19	12(63.2)	7(36.8)	0.004	0.983	-	-
No	82	52(63.4)	30(36.6)				
Flexures	101						
Yes	28	16 (57.1)	12 (42.9)	0.646	0.421	-	-
No	73	48 (65.8)	25 (34.2)				
**Development of PSA**	101						
Yes	31	18(58.1)	13(41.9)	0.543	0.462	-	-
No	70	46(65.7)	24(34.3)				

**Comorbidities**	101						
Yes	57	35(61.4)	22(38.6)	0.217	0.641	-	-
No	44	29(65.9)	15(34.1)				
**Age of onset of MTX**	101	46.05±14.44	44.84±15.56	-	0.701	-	-
**MTX therapy duration (months)**	101	12.0 (5.0-24.3)	18.0 (5.0-41.0)	-	0.219	-	-
**MTX Administration**	101						
Oral	47	29 (61.7)	18 (38.3)	0.779	0.677	-	-
Subcutaneous	30	18 (60.0)	12 (40.0)				
Both	24	17 (70.8)	7 (29.2)				
**Type of MTX therapy**	101						
Monotherapy	93	59 (63.4)	34 (36.6)	0.003	0.958	-	-
Combination Therapy	8	5 (62.5)	3 (37.5)				
**Maximum MTX dose (mg/week)**	101	13.8 (10.0-15.0)	12.5 (10.0-15.0)	-	0.977	-	-
**Therapeutic adherence**							
Adherent	70	46 (65.7)	24 (34.3)	-	0.403*	-	-
Intentional non-adherent	30	18 (60.0)	12 (40.0)				
Unintentional non-adherent	1	0 (0.0)	1 (100.0)				

*p-value for the Fisher's test. PS: psoriasis; PSA: psoriatic arthritis
