# Supplementary material for: ABCC1, ABCG2 and FOXP3: Predictive Biomarkers of Toxicity from Methotrexate Treatment in Patients Diagnosed with Moderate-to-Severe Psoriasis
Source: Biomedicines. 2023 Sep 19;11(9):2567. doi: 10.3390/biomedicines11092567 (PMC10526923; doi:10.3390/biomedicines11092567)
Supplement: Supplementary file 1 [file biomedicines-11-02567-s001.zip › Table S5. Clinical variables and gastrointestinal toxicity.pdf]

Table S5. Clinical variables and gastrointestinal toxicity.

Characteristics	N	Gastrointestinal toxicity		X^2	p-valor	OR	$IC_{95\%}$
		NO N (%)	YES (Grade 1-4) N (%)				
**Gender**	101						
Female	52	32(61.5)	20(38.5)	4.977	**0.026**	**2.78**	**1.14-7.20**
Male	49	40(81.6)	9(18.4)			**1**	**-**
**Age diagnosis PS**	101	29.1 (19.2-45.6)	23.8 (13.8-40.5)	-	0.247	-	-
**Family History of Ps**	101						
Yes	52	37 (71.2)	15 (28.8)	0.001	0.976	-	-
No	49	35 (71.4)	14 (28.6)				
**Smoking**	101						
Smoker	31	26 (83.9)	5 (16.1)	8.259	**0.016**	**1**	**-**
Non-smoking	49	36 (73.5)	13 (26.5)			**1.88**	**0.62-6.45**
Former Smoker	21	10 (47.6)	11 (52.4)			**5.72**	**1.66-22.34**
**Alcoholic drinking**	101						
Drinker	38	33 (86.8)	5 (13.2)	-	**0.013***	**1**	**-**
Non-drinker	61	38 (62.3)	23 (37.7)			**3.99**	**1.46-12.96**
Former Drinker	2	1 (50.0)	1 (50.0)			**6.60**	**0.24-186.55**
**Type of Psoriasis**	101						
Plaque	74	57(77.0)	17(33.0)	-	0.105*	-	-
Pustular	5	2(40.0)	3(60.0)				
Inverse	1	1(100.0)	0(0.0)				
Guttate	5	2(40.0)	3(60.0)				
Plaque and guttate	12	6(50.0)	6(50.0)				
Plaque and inverse	2	2(100.0)	0(0.0)				
Plaque and pustular	1	1(100.0)	0(0.00)				
Plaque, guttate and inverse	1	1(100.0)	0(0.0)				
**Localization**							
Trunk and limbs	101						
Yes	93	65(69.9)	28(30.1)	-	0.433*	-	-
No	8	7(87.5)	1(12.5)				
Scalp and face	101						
Yes	77	53(68.8)	24(31.2)	0.955	0.329	-	-
No	24	19(79.2)	5(20.8)				
Nails	101						
Yes	58	37(63.8)	21(36.2)	3.738	**0.053**	**2.48**	**1.00-6.64**
No	43	35(81.4)	8(18.6)			**1**	**-**
Palmoplantar	101						
Yes	19	14(73.7)	5(26.3)	0.066	0.798	-	-
No	82	58(70.7)	24(29.3)				
Flexures	101						
Yes	28	23 (82.1)	5 (17.9)	2.230	0.135	**-**	**-**
No	73	49 (67.1)	24 (32.9)				
**Development of PSA**	101						
Yes	31	20(64.5)	11(35.5)	1.002	0.317	**-**	**-**
No	70	52(74.3)	18(25.7)				
**Comorbidities**	101						

Yes	57	41(71.9)	16(29.5)	0.026	0.871	-	-
No	44	31(70.5)	13(29.5)				
**Age of onset of MTX**	101	45.92±15.07	44.83±14.32	-	0.735	-	-
**MTX therapy duration (months)**	101	15.0 (5.8-29.5)	12.0 (5.0-38.0)	-	0.559	**-**	**-**
**MTX Administration**	101						
Oral	47	40 (85.1)	7 (14.9)	14.734	**0.001**	**1**	**-**
Subcutaneous	30	22 (73.3)	8 (26.7)			**2.08**	**0.66-6.68**
Both	24	10 (41.7)	14 (58.3)			**8.00**	**2.65-26.52**
**Type of MTX therapy**	101						
Monotherapy	93	66 (71.0)	27 (29.0)	0.059	0.809	-	-
Combination Therapy	8	6 (75.0)	2 (25.0)				
**Maximum MTX dose (mg/week)**	101	15.0 (10.0-15.0)	12.5 (10.0-15.0)	-	0.585	-	-
**Therapeutic adherence**	101						
Adherent	70	53 (75.7)	17 (24.3)	-	0.202*	-	-
Intentional non-adherent	30	18 (60.0)	12 (40.0)				
Unintentional non-adherent	1	1 (100.0)	0 (0.0)				

*p-value for the Fisher's test. PS: psoriasis; PSA: psoriatic arthritis
