# Supplementary material for: ABCC1, ABCG2 and FOXP3: Predictive Biomarkers of Toxicity from Methotrexate Treatment in Patients Diagnosed with Moderate-to-Severe Psoriasis
Source: Biomedicines. 2023 Sep 19;11(9):2567. doi: 10.3390/biomedicines11092567 (PMC10526923; doi:10.3390/biomedicines11092567)
Supplement: Supplementary file 1 [file biomedicines-11-02567-s001.zip › Table S6. Clinical variables and asthenia.pdf]

Table S6. Clinical variables and asthenia

Characteristics	N	Asthenia		X^2	p-valor	OR	$IC_{95\%}$
		NO<br>N (%)	YES<br>(Grade 1-4)<br>N (%)				
**Gender**	101						
Female	52	34(65.4)	18(34.6)	2.541	0.111	-	-
Male	49	39(79.6)	10(20.4)				
**Age diagnosis PS**	101	27.0<br>(19.0-45.8)	29.2<br>(15.8-41.7)	-	0.635	-	-
**Family History of Ps**	101						
Yes	52	38 (73.1)	14 (26.9)	0.034	0.853	-	-
No	49	35 (71.4)	14 (28.6)				
**Smoking**	101						
Smoker	31	23 (74.2)	8 (25.8)	0.421	0.810	-	-
Non-smoking	49	36 (73.5)	13 (26.5)				
Former Smoker	21	14 (66.7)	7 (33.3)				
**Alcoholic drinking**	101						
Drinker	38	31 (81.6)	7 /18.4)	-	0.198*	-	-
Non-drinker	6	41 (67.2)	20 (32.8)				
Former Drinker	2	1 (50.0)	1 (50.0)				
**Type of Psoriasis**	101						
Plaque	74	57(77.0)	17(23.0)	-	0.340*	-	-
Pustular	5	3(60.0)	2(40.0)				
Inverse	1	1(100.0)	0(0.0)				
Guttate	5	2(40.0)	3(60.0)				
Plaque and guttate	12	7(58.3)	5(41.7)				
Plaque and inverse	2	1(50.0)	1(50.0)				
Plaque and pustular	1	1(100.0)	0(0.0)				
Plaque, guttate and inverse	1	1(100.0)	0(0.0)				
**Localization**							
Trunk and limbs	101						
Yes	93	67(72.0)	26(28.0)	-	1*	-	-
No	8	6(75.0)	2(25.0)				
Scalp and face	101						
Yes	77	54(70.1)	23(29.9)	0.746	0.388	-	-
No	24	19(79.2)	5(20.8)				
Nails	101						
Yes	58	40(69.0)	18(31.0)	0.746	0.388	-	-
No	43	33(76.7)	10(23.3)				
Palmoplantar	101						
Yes	19	14(73.7)	5(26.3)	0.023	0.879	-	-
No	82	59(72.0)	23(28.0)				
Flexures	101						
Yes	28	17 (60.7)	11 (39.3)	2.585	0.108	-	-
No	73	56 (76.7)	17 (23.3)				
**Development of PSA**	101						
Yes	31	19(61.3)	12(38.7)	2.695	0.101	-	-
No	70	54(77.1)	16(22.9)				

**Comorbidities**	101						
Yes	57	39(68.4)	18(31.6)	-	0.375*	-	-
No	44	34(77.3)	10(22.7)				
**Age of onset of MTX**	101	45.67±15.60	45.43±12.70	-	0.936	-	-
**MTX therapy duration (months)**	101	12.0 (6.0-29.0)	16.0 (4.0-36.3)	-	0.467	**-**	**-**
**MTX Administration**	101						
Oral	47	42 (89.4)	5 (10.6)	15.693	**<0.001**	**1**	**-**
Subcutaneous	30	20 (66.7)	10 (33.3)			**4.20**	**1.31-15.04**
Both	24	11 (45.8)	13 (54.2)			**9.93**	**3.07-36.90**
**Type of MTX therapy**	101						
Monotherapy	93	68 (73.1)	25 (26.9)	0.415	0.519	-	-
Combination Therapy	8	5 (62.5)	2 (37.5)				
**Maximum MTX dose (mg/week)**	101	12.5 (10.0-15.0)	15.0 (10.0-15.6)	-	0.114	-	-
**Therapeutic adherence**							
Adherent	70	53 (75.7)	17 (24.3)	-	0.444*	-	-
Intentional non-adherent	30	19 (63.3)	11 (36.7)				
Unintentional non-adherent	1	1 (100.0)	0 (0.0)				
*p-value for the Fisher's test. PS: psoriasis; PSA: psoriatic arthritis							
