# Supplementary material for: ABCC1, ABCG2 and FOXP3: Predictive Biomarkers of Toxicity from Methotrexate Treatment in Patients Diagnosed with Moderate-to-Severe Psoriasis
Source: Biomedicines. 2023 Sep 19;11(9):2567. doi: 10.3390/biomedicines11092567 (PMC10526923; doi:10.3390/biomedicines11092567)
Supplement: Supplementary file 1 [file biomedicines-11-02567-s001.zip › Table S7. Clinical variables and neurotoxicity.pdf]

Table S7. Clinical variables and neurotoxicity

Characteristics	N	Neurotoxicity		X^2	p-valor	OR	$IC_{95\%}$
		NO N (%)	SI (Grado 1-4) N (%)				
**Gender**	101						
Female	52	46(88.5)	6(11.5)	-	0.489*	-	-
Male	49	46(93.9)	3(6.1)				
**Age diagnosis PS**	101	27.1 (18.3-43.6)	35.2 (20.9-35.3)	-	0.557	-	-
**Family History of Ps**	101						
Yes	52	49 (94.2)	3 (5.8)	-	0.309*	-	-
No	49	43 (87.8)	6 (12.2)				
**Smoking**	101						
Smoker	31	28 (90.3)	3 (9.7)	-	1*	-	-
Non-smoking	49	45 (91.8)	4 (8.2)				
Former Smoker	21	19 (90.5)	2 (9.5)				
**Alcoholic drinking**	101						
Drinker	38	34 (89.5)	4 (10.5)	-	0.776*	-	-
Non-drinker	61	56 (91.8)	5 (8.2)				
Former Drinker	2	2 (100.0)	0 (0.0)				
**Type of Psoriasis**	101						
Plaque	74	66(89.2)	8(10.8)	-	0.700*	-	-
Pustular	5	5(100.0)	0(0.0)				
Inverse	1	1(100.0)	0(0.0)				
Guttate	5	4(80.0)	1(20.0)				
Plaque and guttate	12	12(100.0)	0(0.0)				
Plaque and inverse	2	2(100.0)	0(0.0)				
Plaque and pustular	1	1(100.0)	0(0.0)				
Plaque, guttate and inverse	1	1(100.0)	0(0.0)				
**Localization**							
Trunk and limbs	101						
Yes	93	86(92.5)	7(7.5)	-	0.149*	-	-
No	8	6(75.0)	2(25.0)				
Scalp and face	101						
Yes	77	70(90.9)	7(9.1)	-	1*	-	-
No	24	22(91.7)	2(8.3)				
Nails	101						
Yes	58	51(87.9)	7(12.1)	-	0.295*	-	-
No	43	41(95.3)	2(4.7)				
Palmoplantar	101						
Yes	19	17(89.5)	2(10.5)	-	0.676*	-	-
No	82	75(91.5)	7(8.5)				
Flexures	101						
Yes	28	22 (78.6)	6 (21.4)	7.479	**0.006**	**6.36**	**1.55-32.17**
No	73	70 (95.9)	3 (4.1)			**1**	**-**
**Development of PSA**	101						
Yes	31	28(90.3)	3(9.7)	-	1*	-	-
No	70	64(91.4)	6(8.6)				
**Comorbidities**	101						

Yes	57	50(87.7)	7(12.3)	-	0.292*	-	-
No	44	42(95.5)	2(4.5)				
**Age of onset of MTX**	101	45.92±14.88	42.33±14.32	-	0.491	-	-
**MTX therapy duration (months)**	101	14.5 (5.0-31.0)	17.0 (7.0-36.0)	-	0.569	**-**	**-**
**MTX Administration**	101						
Oral	47	46 (97.9)	1 (2.1)	-	**0.047***	**1**	**-**
Subcutaneous	30	26 (86.7)	4 (13.3)			**7.07**	**0.98-142.41**
Both	24	20 (83.3)	4 (16.7)			**9.19**	**1.26-186.15**
**Type of MTX therapy**	101						
Monotherapy	93	84 (90.3)	9 (9.7)	-	1*	-	-
Combination Therapy	8	8 (100.0)	0 (0.0)				
**Maximum MTX dose (mg/week)**	101	12.5 (10.0-15.0)	15.0 (10.0-15.0)	-	0.679*	-	-
**Therapeutic adherence**	101						
Adherent	70	64 (91.4)	6 (8.6)	-	1*	-	-
Intentional non-adherent	30	27 (90.0)	3 (10.0)				
Unintentional non-adherent	1	1 (100.0)	0 (0.0)				

*p-value for the Fisher's test. PS: psoriasis; PSA: psoriatic arthritis
