# Supplementary material for: ABCC1, ABCG2 and FOXP3: Predictive Biomarkers of Toxicity from Methotrexate Treatment in Patients Diagnosed with Moderate-to-Severe Psoriasis
Source: Biomedicines. 2023 Sep 19;11(9):2567. doi: 10.3390/biomedicines11092567 (PMC10526923; doi:10.3390/biomedicines11092567)
Supplement: Supplementary file 1 [file biomedicines-11-02567-s001.zip › Table S8. Clinical variables and skin toxicity..pdf]

Table S8. Clinical variables and skin toxicity.

Characteristics	N	Skin toxicity NO N (%)	Skin toxicity YES (Grade 1-4) N (%)	X^2	p-valor	OR	$IC_{95\%}$
**Gender**	101						
Female	52	46(88.5)	6(11.5)	-	0.271*	-	-
Male	49	47(95.9)	2(4.1)				
**Age diagnosis PS**	101	27.0 (18.4-44.3)	29.3 (19.5-35.5)	-	0.667	-	-
**Family History of Ps**	101						
Yes	52	49 (94.2)	3 (5.8)	-	0.479*	-	-
No	49	44 (89.8)	5 (10.2)				
**Smoking**	101						
Smoker	31	28 (90.3)	3 (9.7)	-	0.707*	**-**	**-**
Non-smoking	49	46 (93.9)	3 (6.1)				
Former Smoker	21	19 (90.5)	2 (9.5)				
**Alcoholic drinking**	101						
Drinker	38	36 (94.7)	2 (5.3)	-	0.752*	-	-
Non-drinker	61	55 (90.2)	6 (9.8)				
Former Drinker	2	2 (100.0)	0 (0.0)				
**Type of Psoriasis**	101						
Plaque	74	68(91.9)	6(8.1)	-	0.311*	-	-
Pustular	5	5(100.0)	0(0.0)				
Inverse	1	1(100.0)	0(0.0)				
Guttate	5	3(60.0)	2(40.0)				
Plaque and guttate	12	12(100.0)	0(0.0)				
Plaque and inverse	2	2(100.0)	0(0.0)				
Plaque and pustular	1	1(100.0)	0(0.0)				
Plaque, guttate and inverse	1	1(100.0)	0(0.0)				
**Localization**							
Trunk and limbs	101						
Yes	93	86(92.5)	7(7.5)	-	0.496*	-	-
No	8	7(87.5)	1(12.5)				
Scalp and face	101						
Yes	77	72(93.5)	5(6.5)	-	0.391*	-	-
No	24	21(87.5)	3(12.5)				
Nails	101						
Yes	58	54(93.1)	4(6.9)	-	0.720*	**-**	**-**
No	43	39(90.7)	4(9.3)				
Palmoplantar	101						
Yes	19	19(100.0)	0(0.0)	-	0.346*	-	-
No	82	74(90.2)	8(9.8)				
Flexures	101						
Yes	28	25 (89.3)	3 (10.7)	0.415	0.519	**-**	**-**
No	73	68 (93.2)	5 (6.8)				
**Development of PSA**	101						
Yes	31	25(80.6)	6(19.4)	-	**0.009***	**8.16**	**1.75-58.25**
No	70	68(97.1)	2(2.9)			**1**	**-**
**Comorbidities**	101						

Yes	57	52(91.2)	5(8.8)	-	1*	-	-
No	44	41(93.2)	3(6.8)				
**Age of onset of MTX**	101	45.77±15.18	43.63±9.64	-	0.579	-	-
**MTX therapy duration (months)**	101	15.0 (5.0-33.0)	5.0 (3.3-22.3)	-	0.487	-	-
**MTX Administration**	101						
Oral	47	45 (95.7)	2 (4.3)	-	0.355*	-	-
Subcutaneous	30	26 (86.7)	4 (13.3)				
Both	24	22 (91.7)	2 (8.3)				
**Type of MTX therapy**	101						
Monotherapy	93	86 (92.5)	7 (7.5)	0.249	0.617	-	-
Combination Therapy	8	7 (87.5)	1 (12.5)				
**Maximum MTX dose (mg/week)**	101	12.5 (10.0-15.0)	15.0 (10.0-15.0)	-	0.993	-	-
**Therapeutic adherence**							
Adherent	70	64 (91.4)	6 (8.6)	-	1*	-	-
Intentional non-adherent	30	28 (93.3)	2 (6.7)				
Unintentional non-adherent	1	1 (100.0)	0 (0.0)				

*p-value for the Fisher's test. PS: psoriasis; PSA: psoriatic arthritis
