# Supplementary material for: ABCC1, ABCG2 and FOXP3: Predictive Biomarkers of Toxicity from Methotrexate Treatment in Patients Diagnosed with Moderate-to-Severe Psoriasis
Source: Biomedicines. 2023 Sep 19;11(9):2567. doi: 10.3390/biomedicines11092567 (PMC10526923; doi:10.3390/biomedicines11092567)
Supplement: Supplementary file 1 [file biomedicines-11-02567-s001.zip › Table S9. Clinical variables and infections.pdf]

Table S9. Clinical variables and infections

Characteristics	N	Infections		X^2	p-valor	OR	$IC_{95\%}$
		NO N (%)	YES (Grade 1-4) N (%)				
**Gender**	101						
Female	52	47(90.4)	5(9.6)	-	*0.206	-	-
Male	49	48(98.0)	1(2.0)				
**Age diagnosis PS**	101	27.3 (18.9-43.4)	27.4 (11.3-46.5)	-	0.864	-	-
**Family History of Ps**	101						
Yes	52	47 (90.4)	5 (9.6)	-	0.206*	-	-
No	49	48 (98.0)	1 (2.0)				
**Smoking**	101						
Smoker	31	30 (96.8)	1 (3.2)	-	0.287*	-	-
Non-smoking	49	47 (95.9)	2 (4.1)				
Former Smoker	21	18 (85.7)	3 (14.3)				
**Alcoholic drinking**	101						
Drinker	38	37 (97.4)	1 (2.6)	-	0.471*	-	-
Non-drinker	61	56 (91.8)	5 (8.2)				
Former Drinker	2	2 (100.0)	0 (0.0)				
**Type of Psoriasis**	101						
Plaque	74	69(93.2)	5(6.8)	-	1*	-	-
Pustular	5	5(100.0)	0(0.0)				
Inverse	1	1(100.0)	0(0.0)				
Guttate	5	5(100.0)	0(0.0)				
Plaque and guttate	12	11(91.7)	1(8.3)				
Plaque and inverse	2	2(100.0)	0(0.0)				
Plaque and pustular	1	1(100.0)	0(0.0)				
Plaque, guttate and inverse	1	1(100.0)	0(0.0)				
**Localization**							
Trunk and limbs	101						
Yes	93	88(94.6)	5(5.4)	-	0.399*	-	-
No	8	7(87.5)	1(12.5)				
Scalp and face	101						
Yes	77	75(97.4)	2(2.6)	-	**0.027***	**1**	**-**
No	24	20(83.3)	4(16.7)			**7.50**	**1.36-56.97**
Nails	101						
Yes	58	54(93.1)	4(6.9)	-	1*	-	-
No	43	41(95.3)	2(4.7)				
Palmoplantar	101						
Yes	19	19(100.0)	0(0.0)	-	0.591*	-	-
No	82	76(92.7)	6(7.3)				
Flexures	101						
Yes	28	26 (92.9)	2 (7.1)	-	0.668*	-	-
No	73	69 (94.5)	4 (5.5)				
**Development of PSA**	101						
Yes	31	27(87.1)	4(12.9)	-	0.069*	-	-
No	70	68(97.1)	2(2.9)				

**Comorbidities**	101						
Yes	57	53(93.0)	4(7.0)	-	0.694*	-	-
No	44	42(95.5)	2(4.5)				
**Age of onset of MTX**	101	45.39±15.07	49.00±9.61	-	0.422	-	-
**MTX therapy duration (months)**	101	15.0 (5.0-32.0)	9.0 (6.5-46.8)	-	0.870	-	-
**MTX Administration**	101						
Oral	47	44 (93.6)	3 (6.4)	-	0.866*	-	-
Subcutaneous	30	29 (96.7)	1 (3.3)				
Both	24	22 (91.7)	2 (8.3)				
**Type of MTX therapy**	101						
Monotherapy	93	88 (94.6)	5 (5.4)	0.669	0.413	-	-
Combination Therapy	8	7 (87.5)	1 (12.5)				
**Maximum MTX dose (mg/week)**	101	15.0 (10.0-15.0)	10.0 (10.0-11.9)	-	0.106	-	-
**Therapeutic adherence**	101						
Adherent	70	66 (94.3)	4 (5.7)	-	1*	-	-
Intentional non-adherent	30	28 (93.3)	2 (6.7)				
Unintentional non-adherent	1	1 (100.0)	0 (0.0)				

*p-value for the Fisher's test. PS: psoriasis; PSA: psoriatic arthritis
